# Liquid-Ordered Phase Formation by Mammalian and Yeast Sterols: A Common Feature With Organizational Differences

**DOI:** 10.3389/fcell.2020.00337

**Published:** 2020-06-12

**Authors:** Alena Khmelinskaia, Joaquim M. T. Marquês, André E. P. Bastos, Catarina A. C. Antunes, Andreia Bento-Oliveira, Silvia Scolari, Gerson M. da S. Lobo, Rui Malhó, Andreas Herrmann, H. Susana Marinho, Rodrigo F. M. de Almeida

**Affiliations:** ^1^Centro de Química Estrutural, Faculdade de Ciências, Universidade de Lisboa, Lisbon, Portugal; ^2^Department of Biology, Molecular Biophysics, Humboldt-Universität zu Berlin, Berlin, Germany; ^3^Faculdade de Ciências, BioISI, Universidade de Lisboa, Lisbon, Portugal

**Keywords:** ergosterol, cholesterol, zymosterol, fluorescence lifetime imaging microscopy, fluorescence spectroscopy, plasma membrane, lipid-lipid interactions, sterol-rich domain

## Abstract

Here, biophysical properties of membranes enriched in three metabolically related sterols are analyzed both *in vitro* and *in vivo*. Unlike cholesterol and ergosterol, the common metabolic precursor zymosterol is unable to induce the formation of a liquid ordered (*l*_o_) phase in model lipid membranes and can easily accommodate in a gel phase. As a result, Zym has a marginal ability to modulate the passive membrane permeability of lipid vesicles with different compositions, contrary to cholesterol and ergosterol. Using fluorescence-lifetime imaging microscopy of an aminostyryl dye in living mammalian and yeast cells we established a close parallel between sterol-dependent membrane biophysical properties *in vivo* and *in vitro*. This approach unraveled fundamental differences in yeast and mammalian plasma membrane organization. It is often suggested that, in eukaryotes, areas that are sterol-enriched are also rich in sphingolipids, constituting highly ordered membrane regions. Our results support that while cholesterol is able to interact with saturated lipids, ergosterol seems to interact preferentially with monounsaturated phosphatidylcholines. Taken together, we show that different eukaryotic kingdoms developed unique solutions for the formation of a sterol-rich plasma membrane, a common evolutionary trait that accounts for sterol structural diversity.

## Introduction

**SCHEME 1:**
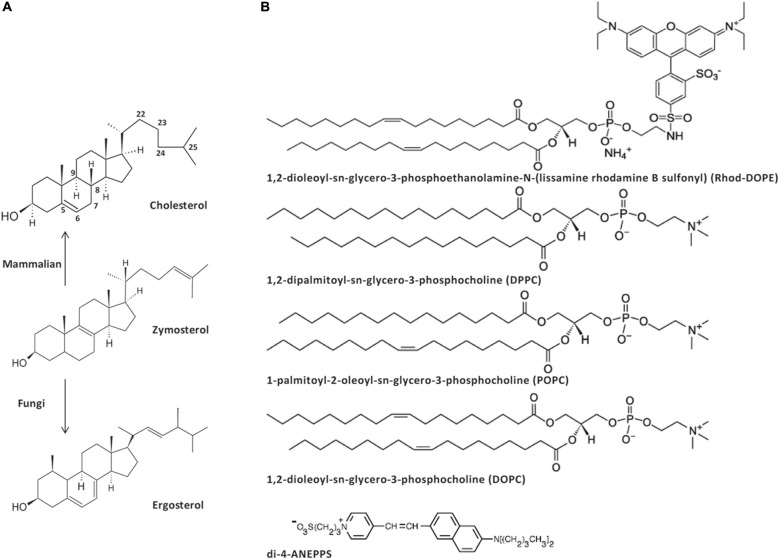
Structure of the sterols **(A)**, phospholipids and dyes **(B)** used in this work. Zymosterol is a biosynthetic precursor of both cholesterol, the major sterol in the plasma membrane of animal cells, and ergosterol, the major sterol in the plasma membrane of fungi.

Ergosterol (Erg) and cholesterol (Chol) are the major sterols in yeast (fungi) and mammalian (animal) cell membrane, respectively. These two sterols share several properties, namely the ability to increase the order of fluid model membranes and to induce the formation of ordered domains when mixed with certain lipids, such as phosphatidylcholines (PCs) with either one or two saturated acyl chains (Ipsen et al., 1990; Vist and Davis, 1990; De Almeida et al., 2003; Silva et al., 2006; Hsueh et al., 2007). Zymosterol (Zym) is a biosynthetic precursor of both Chol and Erg in the Blöch pathway (Megha et al., 2006). Notably, all three molecules have very small structural differences (Scheme 1). Zym is, by proportion, only the fourth main sterol present in the plasma membrane of *Saccharomyces cerevisiae* wild-type (*wt*) cells (Pedroso et al., 2009) but is known to accumulate in certain *S. cerevisiae* mutant strains, such as *erg6*Δ and *erg2*Δ*erg6*Δ cells (Munn et al., 1999; Guan et al., 2009), where its levels raise several-fold becoming a major sterol (Munn et al., 1999; Heese-Peck et al., 2002; Valachovic et al., 2006; Dupont et al., 2011). Despite the changes in sterol profile, the total sterol content in plasma membrane extracts remains essentially unchanged in *erg6*Δ cells (Daum et al., 1999; Abe and Hiraki, 2009; Dupont et al., 2011). Erg and Chol are known to regulate cell membrane permeability (Subczynski et al., 1989; Mukhopadhyay et al., 2002). Yeast cells with deletions in Erg biosynthesis-related genes frequently exhibit increased membrane permeability (Kaur and Bachhawat, 1999; Mukhopadhyay et al., 2002; Folmer et al., 2008) when compared to *wt* cells, which has been attributed to modifications in the sterol profile (Kaur and Bachhawat, 1999), and the consequent altered membrane fluidity (Abe and Hiraki, 2009), or instability of ordered domains (Mukhopadhyay et al., 2002). In fact, Erg and Chol have been considered crucial for the formation of a subset of membrane lipid domains described as being in a liquid ordered (*l*_o_) phase, due to their ability to establish tight interactions with sphingolipids (Simons and Vaz, 2004; Klose et al., 2010). Although both Chol and Erg form *l*_o_ phases, their respective two kingdoms present fundamental differences in their membrane lipid organization, namely the transient nature and nanoscopic scale of the lipid domains in mammalian cells (Sezgin et al., 2017) versus stable large membrane compartments in yeast (Malinsky and Opekarova, 2016; Athanasopoulos et al., 2019; Zahumensky and Malinsky, 2019). Another distinctive feature is the leaflet distribution of either Chol or Erg: whereas for Chol the literature is not consensual in the evidence for its preferential location in the outer/inner leaflet or its even distribution (Steck and Lange, 2018), regarding Erg interleaflet partition recent results point toward a possible preferential location in the inner leaflet (Solanko et al., 2018). Such feature possibly relates with the presence of sphingolipid-enriched gel-like domains, under physiological conditions, in the plasma membrane of yeast (Aresta-Branco et al., 2011). So far, the ability of Zym to form an *l*_o_ phase has not been directly assessed and its biophysical properties are still poorly understood, both *in vitro* and *in vivo*. Yet, it is known that yeast strains defective in Erg biosynthesis, e.g., *erg2*Δ, *erg6*Δ, or *erg2*Δ*erg6*Δ are sensitive to weak organic acids (Mollapour et al., 2004) and a wide variety of drugs (Kaur and Bachhawat, 1999; Emter et al., 2002) to which increased passive diffusion across the plasma membrane is, most likely, determinant (Emter et al., 2002). Consequently, a comprehensive characterization of Zym biophysical properties can help understanding the underlying causes for Erg and Chol selection as the major sterols, respectively, in fungi and animals.

The present work aimed at assessing the ability of metabolically related sterols (Chol, Erg, and Zym) to form *l*_o_ lipid domains and at gaining further insight into the lipid composition of such domains in living cells. We found that, unlike Chol and Erg, Zym can be incorporated to similar extents in both gel and fluid phase PC bilayers without significantly compromising gel-phase dense packing, as shown by the maintenance of a very low permeability, and its inability to efficiently form an *l*_o_ phase. Thus, we hypothesize that *l*_o_ phase formation ability of fungi and mammalian sterols is a convergent evolutionary trait that results in their selective enrichment over Zym (and possibly other sterol precursors). However, whereas in mammalian cells there is a clear biophysical similarity to sphingomyelin (or saturated PC)/chol model membranes, it seems that in *S. cerevisiae* cells sterol-enriched membrane domains bear more resemblance with monounsaturated PC/Erg model mixtures. This might explain fundamental differences between mammalian and fungal cell membrane organization.

## Materials and Methods

### Chemicals and Yeast Strains

1,2-dipalmitoyl-*sn*-glycero-3-phosphocholine (DPPC), 1,2-dioleoyl-*sn*-glycero-3-phosphocholine (DOPC), 1-palmitoyl-2-oleoyl-*sn*-glycero-3-phosphocholine (POPC), and N-(lissamine Rhodamine B sulfonyl)-1,2-dioleoyl-*sn*-3-phosphatidylethanolamine (Rhod-DOPE) were purchased from Avanti Polar Lipids (Alabaster, AL, United States). Chol, Erg, Ludox (colloidal silica diluted to 50 weight% in water), CCCP (carbonyl cyanide *m*-chlorophenyl hydrazine), FCCP (carbonyl cyanide-4-(trifluoromethoxy)phenylhydrazone) and pyranine were purchased from Sigma-Aldrich (St. Louis, MO, United States). Zym was purchased from Avanti or from Steraloids, yielding the same results. Di-4-ANEPPS was purchased from Invitrogen (Madrid, Spain). Valinomycin was purchased from EMD Chemical Inc. (San Diego, CA, United States). Yeast extract, bactopeptone, yeast nitrogen base, and agar were obtained from Difco (Detroit, MI, United States). Solvents for lipid and probe stock solutions were spectroscopic grade. Other reagents were of the highest purity available.

*Saccharomyces cerevisiae* BY4741 (*wt*) cells (genotype *MATa his3*Δ1 *leu2*Δ0 *met15*Δ0 *ura3*Δ0) and *erg6*Δ cells (BY4741*; MATa his3*Δ1 *leu2*Δ0 *met15*Δ0 *ura3*Δ0 *YML008c:*kanMX4) were obtained from EUROSCARF (Frankfurt, Germany). Chinese Hamster Ovary (CHO)-K1 cells were obtained from American Type Culture Collection.

### Media and Growth Conditions

*Saccharomyces cerevisiae* cells were cultured as described previously (Aresta-Branco et al., 2011). Briefly, cells were grown overnight at 30°C in synthetic complete medium (SC) containing 2% (w/v) glucose, 0.68% (w/v) yeast nitrogen base and amino acids as indicated in Pedroso et al. (2009). Cells were inoculated at an A_600_ of 0.15 and, after incubation for 5–6 h, cells in mid-exponential phase were harvested at an A_600_ = 0.6.

CHO-K1 cells were grown in Dulbecco’s modified Eagle’s medium (PAN Biotech, Aidenbach, Germany) without phenol red and supplied with 2 mM L-Glutamine, 10% FBS and 5% PS (complete medium) and incubated at 37°C and 5% CO_2_. About 48 h prior to experiment cells were plated into 35 mm Matek dishes till 80–90% confluence.

### Cell Preparation

Harvested yeast cells (*wt*, *erg6*Δ) were washed twice with sterile water and then suspended in 100 mM sodium dihydrogen phosphate, 100 mM sodium chloride, 1 mM EDTA, pH 7.4 buffer solution (fluorescence buffer). The probe di-4-ANEPPS was added from a concentrated methanol stock solution to the cells at a final concentration of 1 μM and incubated for 5 min, at room temperature (24°C). After incubation, the cells were centrifuged and resuspended in the fluorescence buffer. For CCCP treatment, cells were incubated with CCCP [20 μM (Grossmann et al., 2007) in buffer] throughout imaging.

CHO-K1 cells were washed twice in fluorescence buffer prior to imaging and treatments. The probe was diluted 10× in fluorescence buffer. Cell staining with 0.1 μM di-4-ANEPPS was then carried out for 5–40 min at 24°C. To allow for intracellular membrane staining the treatment was prolonged to 90 min. Cells were then washed twice with fluorescence buffer prior to imaging. For methyl-β-cyclodextrin (MβCD) treatment, before staining CHO cells were incubated with MβCD (5 mM) for 1 min at 4°C, which removes approximately 12 mol% of Chol from the plasma membrane (Scolari et al., 2009).

### Vesicles Preparation

Multilamellar vesicles (MLVs) containing the appropriate lipids, as well as di-4-ANEPPS when used, were prepared by previously described methods (Bastos et al., 2012). The lipid was hydrated by the addition of 1 mL of HEPES buffer (10 mM, NaCl 150 mM pH 7.4), previously heated above the main transition temperature (*T*_m_) of the lipids. The MLV suspension was slowly cooled down and kept in the dark at 4°C. Before measurement, the liposomes suspension was slowly brought to room temperature. All the procedures involving Erg and Zym were performed in the dark. Large unilamellar vesicles (LUVs) with ca. 100 nm diameter were obtained by MLV extrusion (Bastos et al., 2012).

Giant unilamellar vesicles (GUVs) were prepared by electroformation in titanium chambers as previously described (Stockl et al., 2010; Marques et al., 2015). The lipid film in the titanium chambers was hydrated with 700 μL of 250 mM sucrose solution, with 15 mM NaN_3_, 280 mOsm/kg in MilliQ water, sealed and connected to the signal generator and subjected to the sequence of signals described in Marques et al. (2015). In case of DPPC-containing mixtures, the chambers were equilibrated with a block heater at 60°C throughout electroformation, and the sucrose buffer was previously heated to that temperature. The vesicle suspension was kept at room temperature (24°C) in the dark until use. The osmolality of the solutions used in GUV preparation/observation was checked with a 3300 cryo-osmometer (Advanced Instruments, Norwood, MA, United States).

DPPC, DOPC and POPC concentrations in stock solutions (chloroform) were determined by phosphorus analysis (McClare, 1971). Probe concentrations were determined spectrophotometrically: ε (Rhod-DOPE, λ_max_ = 559 nm, chloroform) = 95 × 10^3^ M^–1^ cm^–1^; ε(di-4-ANEPPS, λ_max_ = 497 nm, methanol) = 42 × 10^3^ M^–1^ cm^–1^; ε(*t*-PnA, λ_max_ = 300 nm, ethanol) = 89 × 10^3^ M^–1^ cm^–1^ (Johnson and Spence, 2010).

Sterol concentrations in stock solutions (chloroform) were determined by gravimetry.

### Confocal Fluorescence Microscopy of GUVs

An aliquot (100 μL) of GUV suspension was added to a chamber of an eight-well plastic plate with glass-like coverslip bottom (Ibidi GmbH, Germany). A volume of 150 μL of glucose buffer (250 mM glucose, 5.8 mM Na_2_HPO_4_, 5.8 mM NaH_2_PO_4_) 300 mOsm/kg, pH 7.2, in MilliQ water was added, forcing the GUVs to precipitate in the bottom of the chamber. GUVs were observed with a Leica SP-E confocal inverted microscope (Leica Microsistemas, Lisbon, Portugal) using the 488 nm excitation line at <20% laser intensity and operating in the mode 1024 × 1024, 400 Hz. A ×63 HCX PL APO oil immersion (NA = 1.4) objective (Leica) was used. Thin Z-optical sections (≈ 0.6–0.8 μm thick) were acquired and 3D projections of GUV hemispheres obtained using Leica software. Rhod-DOPE was the probe used to label the GUVs and was co-dissolved with the lipids at a probe/lipid ratio of 1:500. At least three independent GUV preparations were used for each mixture, showing consistent phase behavior among all GUVs and between samples.

### Absorption and Fluorescence Measurements and Data Analysis

Spectrophotometric absorption measurements were performed on a Jasco V-560 (Tokyo, Japan) double beam spectrophotometer.

Fluorescence measurements were carried out on a Horiba Jobin Yvon Flurolog 3.22 spectrofluorometer at 24°C described previously (Aresta-Branco et al., 2011).

The probe/lipid ratio used with di-4-ANEPPS was 1:500, for a total lipid concentration of 0.4 mM and at least three independent samples were analyzed. For steady-state spectra the bandwidths were 4 nm in both excitation and emission. The spectra representing the median are shown. Other details are given with the results.

For time-resolved measurements by the single photon counting technique, a nanoLED N-460 (Horiba Jobin Yvon) was used for the excitation of di-4-ANEPPS, and the emission wavelength was set to 610 nm with a bandwidth of 15 nm. Other conditions were as described in Bastos et al. (2012). Data analysis was performed as previously described (Bastos et al., 2012; Khmelinskaia et al., 2014).

Briefly, a normalized fluorescence intensity decay is described by a sum of exponentials, i.e.,

(1) $$\begin{array}{c} I\,\left(t\right)=\sum_{i=1}^{n}\alpha_{i}\,e\,x\,p\,\left(-t/\tau_{i}\right) \end{array}$$

where α*_*i*_* and τ*_*i*_* are the normalized amplitude and the lifetime of component *i*, respectively. The (intensity-weighted) mean fluorescence lifetime is given by

(2) $$\begin{array}{c} <\tau \geq \sum \alpha_{i}\,\tau_{i}^{2}/\sum \alpha_{i}\,\tau_{i} \end{array}$$

and the amplitude-weighted average lifetime is defined as

(3) $$\begin{array}{c} \bar{\tau }=\sum \alpha_{i}\,\tau_{i} \end{array}$$

Two exponentials were required to describe di-4-ANEPPS decays (plus a scattering factor). All the data represents the mean ± standard deviation of at least three independent samples.

### Confocal Microscopy and FLIM of Living Cells Labeled With di-4-ANEPPS

Intensity images as well as fluorescence lifetime imaging microscopy (FLIM) measurements were taken in the confocal mode using an Olympus Fluoview, 1000 (Olympus, Tokyo, Japan) equipped with a time-resolved LSM upgrade kit (PicoQuant GmbH, Berlin, Germany) and a x60 (1.35 N.A.) UPlanSApo oil immersion objective (Olympus). Di-4-ANEPPS was excited at 488 nm using the corresponding line of a multiline argon laser and was detected in the range of 565–665 nm. Images with a frame size of 512 × 512 pixels were acquired. For FLIM measurements, di-4-ANEPPS was excited at 440 nm using a pulsed-laser diode. Fluorescence was detected by a single photon avalanche photodiode (SPAD) and a 620 ± 30 nm bandpass filter was used. Further details of the experimental settings and data analysis can be found in Supplementary Note 3. For every single cell the average lifetime <τ> and the amplitude weighted lifetime $\bar{\tau }$ were calculated using equations 2 and 3. The values reported in Table 2 are the average of 30–100 cells of at least three independent experiments. The fluorescence lifetime of the probe was independent of the incubation time. The quality of the fitting was judged by the distribution of the residuals and the χ^2^ value.

### Water Permeability Assay

For the water permeability studies, unlabeled MLVs were prepared in Tris–HCl 5 mM, pH 7.0, 100 mM sucrose buffer (isotonic buffer). The liposomes contained an additional 4 mol% of dicetylphosphate (Sigma-Aldrich), to stabilize the suspension (Bittman and Blau, 1976).

Isotonic MLV suspensions were diluted approximately 1/20 (0.123 mL added to 2.5 mL) in hypotonic buffer (Tris–HCl 5 mM, pH 7.0). The transfer of MLVs from an isotonic sucrose solution into a hypotonic solution, accompanied by a reduction of the suspension turbidity, was monitored overtime by measuring the time dependence of absorbance at 550 nm (Bittman and Blau, 1976) for 5 min (Jasco V-560, Easton, MD, United States). All the data represents the mean ± standard deviation of at least three independent samples. For more details see Supplementary Note 1 and Supplementary Figure S2.

### K^+^/H^+^ Exchange Assay

LUV of equimolar DPPC/DOPC and DPPC/DOPC/sterol mixtures were prepared in HEPES 20 mM, K_2_SO_4_ 100 mM, pH 7.4 with 0.5 mM pyranine (K-Pyr-buffer). The excess pyranine was removed by gel filtration in a Sephadex G-25 column equilibrated in K-buffer (HEPES 20 mM, K_2_SO4 100 mM, pH 7.4 buffer) and the LUVs with encapsulated pyranine were collected.

The effect of sterol on the potassium permeability of the lipid vesicles was monitored by measuring the variation in fluorescence intensity of membrane impermeable pyranine, as described elsewhere (Coutinho et al., 2004). Further experimental details can be found under Supplementary Note 2 and Supplementary Figure S3. For a quantitative comparison between sterols, the initial slope of the curves was calculated, as a measure of bilayer permeability to potassium (Table 1). All the data represents the mean ± standard deviation of at least four independent samples. Only samples with the same final gradient were selected for analysis, i.e., with the same qualitative behavior of fluorescence intensity and similar final relative emission intensity.

**TABLE 1:** Zym is unable to regulate the passive permeability of lipid bilayers in contrast to Erg and Chol.

	Δ(1/A _550 nm_)/min		IF_510 nm_ (a.u.)/min
Sterol	DPPC	DPPC/DOPC	DPPC/DOPC
–	0.022 ± 0.001	0.187 ± 0.011	−0.263 ± 0.035
Erg	0.120 ± 0.001	0.081 ± 0.012	−0.091 ± 0.031
Zym	0.023 ± 0.005	0.155 ± 0.012	−0.157 ± 0.028
Chol	0.062 ± 0.001	0.082 ± 0.007	−0.074 ± 0.021

*Passive permeability to water is expressed as a variation of MLV suspension absorbance at 550 nm [Δ(1/A_550 nm_)/min] when transferred to hypotonic buffer. Passive permeability to potassium/proton is expressed as a variation of pyranine fluorescence intensity at 510 nm over time [IF_510 nm_(a.u.)/min]. Each sterol was added in equimolar proportion to the lipid mixture.*

## Results

### Zym Is Unable to Support Liquid Ordered/Liquid Disordered Phase Coexistence

In order to directly evaluate the ability of Zym to induce the coexistence of liquid/liquid domains, Erg or Zym were incorporated into GUVs containing equimolar ratios of the saturated lipid DPPC and the unsaturated lipid DOPC, and their behaviors were compared to that of the DOPC/DPPC binary mixture. DOPC/DPPC/Chol system labeled with the same fluorescent lipid analog, Rhod-DOPE, has been previously studied in detail (De Almeida et al., 2007). This probe has been shown to preferentially label the liquid disordered (*l*_d_) phase versus gel (De Almeida et al., 2007), Chol-induced *l*_o_ (De Almeida et al., 2005), and Erg-induced *l*_o_ (Bastos et al., 2012) domains.

**FIGURE 1:**
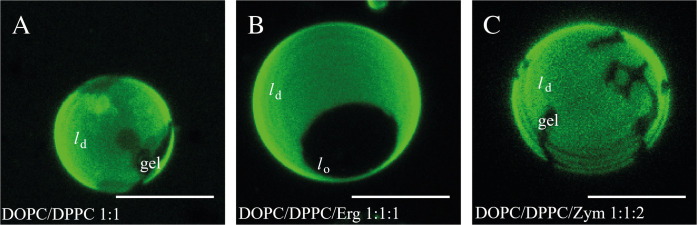
Zym does not promote the formation of coexisting *l*_d_/*l*_o_ liquid phases. Confocal microscopy images of GUVs of **(A)** DOPC/DPPC (1:1 molar ratio), **(B)** DOPC/DPPC/Erg (1:1:1 molar ratio), **(C)** DOPC/DPPC/Zym (1:1:2 molar ratio) at 23°C, labeled with Rhod-DOPE (0.2 mol%). Scale bar = 5 μm.

For the binary equimolar mixture of DPPC and DOPC, the confocal microscopy images showed a Rhod-DOPE distribution with bright, unsaturated lipid-enriched and dark, saturated lipid-enriched domains (Figure 1A). In this binary mixture the domains observed correspond to gel (dark) and *l*_d_ (bright) phase coexistence, with relative areas in agreement with the phase diagram for this mixture (De Almeida et al., 2007). The domains are irregularly shaped, as characteristic for gel/fluid coexistence, in contrast to *l*_d_/*l*_o_ phase coexistence which leads to round-shaped domains (Dietrich et al., 2001). In addition, if Chol is added to form a ternary 1:1:2 (molar ratio) mixture, the vesicles are completely in *l*_o_ state, whereas for the 1:1:1 equimolar proportion it is well known that *l*_d_/*l*_o_ phase separation occurs (Supplementary Figure S1A) (Veatch and Keller, 2003; De Almeida et al., 2007).

In the case of Erg, our results also indicate a clear *l*_d_/*l*_o_ phase coexistence for the lipid composition DOPC/DPPC/Erg (1:1:1, molar ratio) (Figure 1B and Supplementary Figure S1B), as a single dark round *l*_o_ domain is observed. Indeed, GUVs with similar proportions of DOPC/DPPC/Erg have previously been reported to present a bright *l*_d_ phase, rich in unsaturated lipid (DOPC), coexisting with dark *l*_o_ domains, rich in saturated lipid (DPPC) and Erg (Beattie et al., 2005).

For the DOPC/DPPC/Zym (1:1:2, molar ratio) (Figure 1C and Supplementary Figure S1D), non-round gel phase domains, similar to those found in DOPC/DPPC vesicles (Figure 1A), were clearly observed. This indicates that Zym, unlike Chol and Erg, does not have the ability to promote the formation of coexisting *l*_d_/*l*_o_ phases from a pre-existing *l*_d_/gel situation. Since an estimation of the gel area fraction from visual inspection of several GUVs reveals no marked differences on the fraction of gel and *l*_d_ phases with or without Zym, the partition of this sterol among the two phases seems to be similar. Analogous results were obtained for equimolar mixtures (1:1:1 molar ratio) of DOPC/DPPC/Zym (Supplementary Figure S1C).

### Zym Is Unable to Affect the Passive Permeability of Lipid Bilayers

One major difference between gel and *l*_d_ phase with direct and vital biological significance is membrane passive permeability, which is known to be modulated by sterols (Scheidt et al., 2013). Thus, passive permeability to water (Supplementary Note 1 and Supplementary Figure S2) and to ions (Supplementary Note 2 and Supplementary Figure S3) was studied in lipid vesicles formed by binary and ternary mixtures containing each of the metabolically related sterols (DPPC/sterol 2:1 and DPPC/DOPC/sterol 1:1:1 molar ratio, respectively).

A higher permeability to water is expressed as a higher rate of increase of liposomal volume (Table 1). DPPC and DPPC/DOPC (1:1 molar ratio) show opposite behavior: while the highly organized DPPC (gel phase) is quite impermeable to water, the presence of a DOPC-rich fluid phase and the associated packing defects at the interface between coexisting gel and *l*_d_ domains (Clerc and Thompson, 1995) in DPPC/DOPC (1:1 molar ratio) mixtures at room temperature lead to the highest permeability of all the MLVs tested.

The incorporation of Erg or Chol increased the water permeability of the DPPC bilayer (Supplementary Figure S2A and Table 1). Erg had a stronger effect than Chol on bilayer permeability, while Zym had a negligible effect. This typical behavior of Chol and Erg is assigned to the formation of the *l*_o_ phase which, although ordered, has a higher fluidity than the gel phase and a higher passive permeability (Bittman and Blau, 1976).

An opposite effect was observed for DPPC/DOPC mixtures – the incorporation of an equimolar proportion of sterol reduces the water permeability of the bilayer (Supplementary Figure S2B and Table 1). The effect was higher for Erg and Chol in comparison to Zym. Although for binary mixtures the intrinsic bulk permeability of the DPPC-rich phase became higher in the presence of sterol, we found that overall the permeability significantly decreased for DPPC/DOPC membranes containing Chol or Erg. By fluidizing gel domains (Figure 1), the interaction between lipids at the domain interface is facilitated and the mismatch between ordered and disordered phases is reduced, resulting in a decrease of lipid bilayer permeability relatively to gel/*l*_d_ domain interfaces (Cordeiro, 2018; Kirsch and Böckmann, 2019). More specifically, an equimolar proportion of Chol in DPPC/DOPC bilayers corresponds to a *l*_d_/*l*_o_ phase coexistence (De Almeida et al., 2007) and the interface between two liquid phases is much less prone to packing defects than that between a gel and *l*_d_ phase, that would be present in the absence of sterol. The same explanation holds for the observation of a similar effect on permeability when Erg is present in DPPC/DOPC vesicles and is consistent with the *l*_d_/*l*_o_ phase coexistence observed in GUVs (Figure 1). Thus, permeability of domain interfaces is a crucial factor for the total passive permeability in ternary mixtures. The small effect of Zym, as it is incapable of fluidizing the gel phase through *l*_o_ formation, supports that it accommodates well between the tightly packed phospholipids, most probably by adopting a highly planar structure.

Comparable results to those obtained for water permeability were observed for the bilayer permeability to monovalent cations reported by the fluorescence intensity kinetics of the pH-sensitive probe pyranine (Table 1 and Supplementary Figure S3). In DPPC/DOPC mixtures, the incorporation of an equimolar proportion of sterol reduced the K^+^/H^+^ exchange rate, but the effect of the *l*_o_-promoting sterols, Chol and Erg, was much stronger when compared to Zym.

Overall, these results support that the formation of *l*_o_ domains promoted by Erg and Chol is directly related to the control of membrane passive permeability in highly heterogeneous membranes. Additionally, our experiments highlight that *l*_o_ domains, with properties halfway between *l*_d_ and gel, are important for reducing passive permeability at the interfaces between membrane domains.

### Erg, Chol and Zym Distinctly Affect the Dielectric Properties of Phospholipid Bilayers

**FIGURE 2:**
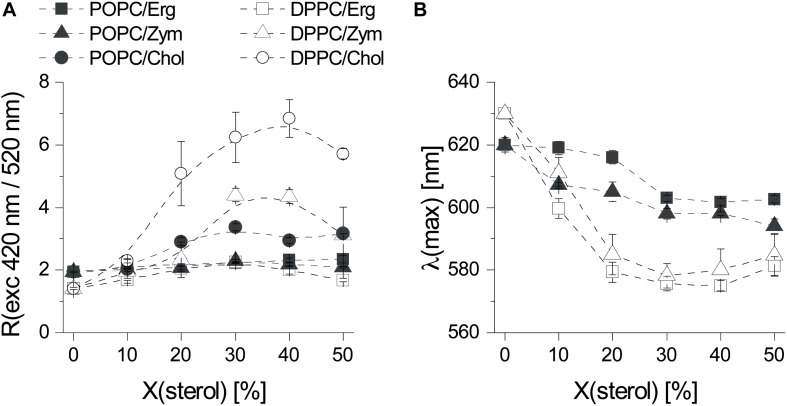
The three sterols Erg, Chol, and Zym induce distinct biophysical changes in phospholipid bilayers. **(A)** Fluorescence excitation ratio, R_ex_, as a function of sterol mole fraction in POPC and DPPC MLVs. R_ex_ is defined as the ratio of fluorescence emission intensity of di-4-ANEPPS arising from excitation at 420 nm divided by the one arising from excitation at 520 nm. **(B)** Influence of Erg and Zym on the polarity at the membrane/water interface expressed as the sterol induced shift of the wavelength of maximum emission intensity of di-4-ANEPPS as a function of sterol mole fraction in POPC/sterol and DPPC/sterol binary mixtures. The values are the mean ± S.D. of at least three independent experiments. Probe:lipid ratio 1:500.

Sterols, in particular Chol, are known to increase the membrane dipole potential when mixed with phospholipids (Haldar et al., 2012). Hence we tested the effect of each sterol on the membrane dipole potential and its connection to *l*_o_ phase. Due to its complex photophysics, di-4-ANEPPS is responsive to changes in polarity or membrane potential and hydration (Loew et al., 1992; Loew, 1996; Amaro et al., 2017). The well-established excitation ratiometric method (Haldar et al., 2012) with this dye was thus used to assess the membrane dipole potential in binary systems (Figure 2A). Representative excitation and emission spectra of di-4-ANEPPS incorporated into the different systems under study are shown in Supplementary Figure S4.

When added to fluid POPC, neither Erg nor Zym had pronounced effects on the dipole potential, whereas Chol induced a modest but noticeable increase (Figure 2A). For Erg and Chol the results are in full agreement with a study performed using a similar probe, di-8-ANEPPS (Haldar et al., 2012). In the presence of a DPPC gel phase, Erg had the lowest effect on membrane dipole potential, whereas Chol affected it the most. Despite differing in the induced membrane potential, both Chol and Erg are consensual *l*_o_-forming/“raft-promoting” sterols. Thus, a very high membrane dipole potential is a particular feature of Chol-enriched domains but cannot be directly related to the *l*_o_ phase forming ability of sterols.

Next, we analyzed the sterol-induced spectral shift of di-4-ANEPPS emission by Erg and Zym (Figure 2B), which relates to the hydration layer and the solvent relaxation in the vicinity of the lipid bilayer (Loew, 1996). For Chol, the spectral shifts obtained are much larger for saturated gel phase lipids, such as DPPC or sphingomyelin, than for unsaturated *l*_d_ phase lipids, such as POPC or DOPC (Bastos et al., 2012). When comparing Erg and Zym in POPC/sterol mixtures, blue-shifts of emission are large even for low Zym, whereas for Erg, the blue-shift is small when *l*_d_ phase still persists (Erg < 30 mol%), increasing more steeply when the system approaches the full *l*_o_ state. This possibly derives from Zym’s homogeneous distribution throughout the membrane, affecting membrane properties globally, whereas the effect of Erg on membrane hydration depends on its concentration and hence the proportion of *l*_d_ to *l*_o_. Although the sterol-induced spectral shift follows the same trend in DPPC mixtures with Erg and Zym, the difference between the shifts in a gel versus an *l*_d_ PC bilayer is larger in the case of Erg. Moreover, the absolute magnitude of the emission blue-shift is generally larger for Chol- than for Erg-containing lipid bilayers (Bastos et al., 2012).

### Zym Is Unable to Discriminate Gel and l_d_ Phases

In a previous study (Bastos et al., 2012) we have shown that di-4-ANEPPS fluorescence properties respond specifically to sterol type and levels, as well as to sterol-phospholipid interactions. Similarly, the fluorescence lifetime of di-4-ANEPPDHQ, a probe of the same family, is considerably higher in lipid vesicles containing Chol (Amaro et al., 2017) and, in addition, the removal of Chol from the plasma membrane of mammalian cells with MβCD leads to the decrease of the lifetime of this probe (Owen et al., 2006). Moreover, these fluorescent probes can be very efficiently excited with visible light with absorption maximum close to the wavelength of common pulsed excitation lasers used in time-resolved fluorescence microscopy, in opposition to other polarity and sterol sensitive dyes e.g., of the family of Laurdan (Golfetto et al., 2013; Mazeres et al., 2017).

**FIGURE 3:**
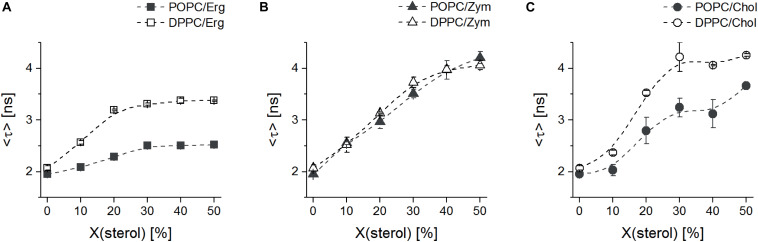
The *l*_o_-promoting sterols Erg and Chol induce clearly distinct changes in lipid properties in saturated (DPPC) versus unsaturated (POPC) phospholipids. Mean fluorescence lifetime <τ> of di-4-ANEPPS in binary mixtures of MLVs with Erg **(A)**, Zym **(B)** or Chol **(C)** and DPPC (open symbols) or POPC (solid symbols), as a function of sterol mole fraction, at 24°C. The values are the mean ± S.D. of at least three independent experiments. Probe:lipid ratio 1:500.

To ensure that di-4-ANEPPS mainly reports on sterol dependent properties, we analyzed its fluorescence intensity decays in LUVs made of binary mixtures comprising a PC, either the fully saturated 16:0 DPPC (gel phase forming) or the mono-unsaturated 16:0,18:1Δ^9^*^*c*^* POPC (*l*_d_ phase forming), and each sterol. The parameters obtained from the analysis of such decays are shown in Table 2. Notably, intensity-weighted mean fluorescence lifetime <τ> of di-4-ANEPPS in saturated lipid (DPPC) or unsaturated lipid (POPC) bilayers devoid of any sterol was similar (≈ 1.8 ns and 1.9 ns, respectively) [Figure 3 and Bastos et al. (2012)]. The results in Figure 3 show that all three sterols increase <τ> of di-4-ANEPPS. This indicates a higher quantum yield of the probe in sterol-enriched phases. Moreover, the membrane/water partition coefficient of di-4-ANEPPS previously determined for the POPC/Erg binary system was higher (∼ 2.1×) for an *l*_o_ membrane than for an *l*_d_ membrane (Bastos et al., 2012) and its quantum yield was found to be larger when incorporated in the *l*_o_ phase (Khmelinskaia et al., 2014).

**TABLE 2:** The molecular environment reported by di-4-ANEPPS is comparable in living cell membranes and model systems containing the same major sterol in a similar fraction.

Sterol	System	α_*1*_	τ_1_ (ns)	α_*2*_	τ_2_ (ns)	$\bar{\tau }$ (ns)	<τ> (ns)
Erg	*S. cerevisiae wt* PM	0.40	1.50	0.60	2.95	2.37	2.58
	POPC/Erg (7:3)	0.41	1.53	0.59	2.77	2.26	2.42
	^#^ DPPC/Erg (7:3)	0.50	1.50	0.50	3.90	2.70	3.24
Zym	*S. cerevisiae erg6*Δ PM	0.60	1.79	0.40	3.60	2.47	2.76
	DPPC/Zym (9:1)	0.65	1.70	0.35	3.51	2.33	2.64
	DPPC/Zym (8:2)	0.50	2.21	0.50	3.86	3.03	3.25
	POPC/Zym (9:1)	0.47	1.90	0.43	3.05	2.32	2.55
	POPC/Zym (8:2)	0.46	2.02	0.54	3.45	2.81	2.98
Chol	CHO-K1 PM	0.27	2.39	0.73	4.65	4.04	4.27
	CHO-K1 IM	0.41	1.99	0.59	4.27	3.35	3.72
	^#^ DPPC/Chol (7:3)	0.23	2.09	0.77	4.50	4.01	4.22
	^#^ PSM/Chol (7:3)	0.24	1.75	0.76	4.74	4.03	4.43
	^#^ POPC/Chol (6:4)	0.43	2.02	0.57	3.57	2.92	3.12

*Fluorescence intensity decay parameters of di-4-ANEPPS incorporated in yeast (*S. cerevisiae*) and mammalian (CHO-K1) cells, and in LUVs of different compositions containing Chol, Erg or Zym, at 24°C. The fluorescence intensity decays measured both in cells by FLIM (30–100 cells in three independent experiments) and in LUVs suspensions by fluorescence spectroscopy (at least three independent experiments) were described by the sum of two exponentials, with amplitudes α_1_ and α_2_ and lifetimes **τ_1_** and **τ_2_**. $\bar{\text{τ}}$ is the amplitude-weighted average fluorescence lifetime and <τ> is the intensity-weighted mean fluorescence lifetime (Eqs. 1–3). ^#^Values from Bastos et al. (2012).*

In the presence of Chol and Zym, <τ> was longer than for the same Erg mole fractions, in agreement with the spectral emission blue-shifts previously discussed (Figure 2B). However, when comparing the results obtained for DPPC and POPC, it is obvious that Chol and Erg have a much stronger effect in the gel phase lipid, since <τ> of di-4-ANEPPS had a higher increase in vesicles with DPPC than with POPC. On the other hand, Zym showed a unique behavior since <τ> obtained in DPPC or POPC vesicles containing this sterol largely overlapped. This observation, in good agreement with the approximately equal surface area of each phase in DOPC:DPPC:Zym and DOPC/DPPC GUVs (Figure 1 and Supplementary Figure S1), suggests that Zym is not able to discriminate gel and *l*_d_ phase phospholipids and seems to be equally well accommodated in both phases. The behavior of di-4-ANEPPS <τ> (Figure 3), regardless of the maximum value reached in each particular case, is similar for the Chol and Erg mixtures with DPPC, reaching a plateau slightly before the system is completely in *l*_o_ phase (≈ 30 mol% sterol) (Sankaram and Thompson, 1991; Hsueh et al., 2005; Silva et al., 2006), which is consistent with a preference of the probe for the sterol-enriched *l*_o_ phase. In contrast, the trend of di-4-ANEPPS <τ> is approximately linear in the case of Zym, without reaching a clear plateau, indicating the absence of a phase separation and of *l*_o_ phase formation. The linear trend up to 40/50 mol% Zym is indicative of the efficient incorporation of this sterol into the membrane, despite the lack of marked changes in domain organization and membrane permeability shown above. It should be noted that, in the case of Erg, the sterol solubility limit might have been transposed, particularly when mixed with POPC. Nonetheless, both DPH (1,6-diphenylhexatriene) fluorescence anisotropy in POPC/Erg liposomes (Silva et al., 2006) and the mean temperature of one broad endotherm component of DPPC/Erg mixtures (Mannock et al., 2010) increase up to 40 mol% of the sterol. Moreover, no Erg crystallites were detected at molar proportions as high as 50 mol% in the same DPPC/Erg mixtures (Mannock et al., 2010). The solubility of Chol in both saturated and monounsaturated phosphocholine bilayers, on the other hand, is unarguably higher than 50 mol% (Huang et al., 1999).

### Sterol Type and Fraction Dictate the Molecular Environment Reported by di-4-ANEPPS in Living Cell Membranes

**FIGURE 4:**
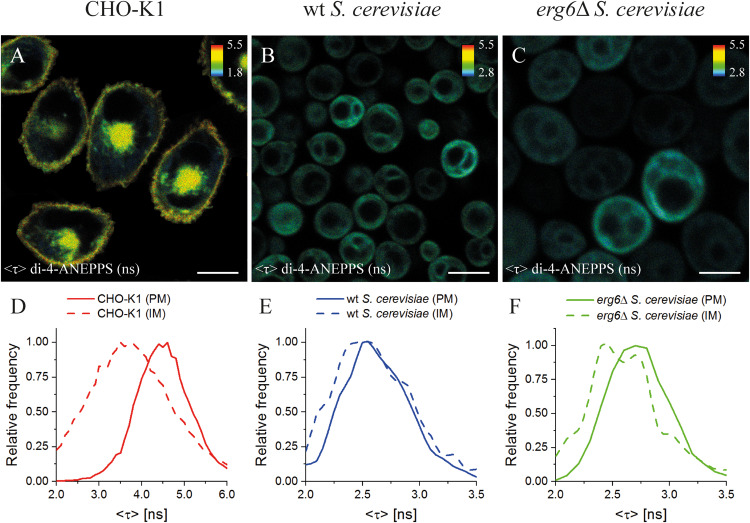
Mean fluorescence lifetime <τ> of di-4-ANEPPS unravels significant differences in lipid membrane organization between mammalian and yeast cells. FLIM images of mammalian (CHO-K1, **A**), *wt* **(B)** and *erg6*Δ **(C)** *S. cerevisiae* cells. The inset color bars give the <τ> range in ns used in the respective image. Scale bar corresponds to 10 μm for **(A,B)**, 5 μm for **(C)**. Frequency histograms of <τ> **(D–F)** corresponding to the plasma membrane, PM (solid lines), or intracellular membrane, IM (dashed lines), for each cell type are shown. The corresponding regions of interest can be found in Supplementary Figure S5.

Finally, we analyzed living systems naturally containing different sterols in their plasma membrane: mammalian CHO-K1 cells (Chol-containing), *wt* (Erg-containing) and *erg6*Δ (Zym-enriched in comparison to *wt*; no Erg) *S. cerevisiae* cells. The single mutant *erg6*Δ was used instead of the double mutant *erg6*Δ*erg2*Δ, with higher levels of Zym, since double mutants in the Erg biosynthetic pathway have a slower growth rate and exhibit a lower total sterol content than their progenitors (Barton et al., 1975). Cells were labeled with di-4-ANEPPS and FLIM experiments were conducted (Figure 4) to analyze both the plasma membrane and the intracellular membranes of individual cells (Supplementary Note 3, Supplementary Figures S5, S6 and Supplementary Table S1). Importantly, since Erg seems to preferentially localize in the inner leaflet (Solanko et al., 2018), di-4-ANNEPS is suitable to probe these Erg-enriched regions not only because, as di-4-ANEPPDHQ, its fluorescence properties are sensitive to medium polarity, membrane potential and hydration, but also because of its ability to flip flop and distribute across both membrane leaflets as a non-charged and short-chained probe, in comparison with the double positively charged di-4-ANEPPDHQ. As the cellular environment is highly complex, to better understand how sterol structure defines biophysical properties of the plasma membrane in eukaryotes, and what simple lipid mixtures may reflect sterol-dependent properties as reported by di-4-ANEPPS fluorescence intensity decay parameters, we compared the cellular results with those in model membranes.

In CHO-K1 cells, a significant difference between the average fluorescence lifetime <τ> of di-4-ANEPPS in the plasma membrane (4.27 ± 0.09 ns) and intracellular membranes (3.72 ± 0.20 ns) was observed (Figures 4A,D and Supplementary Figures S5, S6A, S7D). As <τ> of di-4-ANEPPS is sensitive to sterol levels (see Supplementary Notes 4, 5), this difference of <τ> is most probably due to the lower content of Chol in intracellular membranes than in the plasma membrane, a general feature of mammalian cells (Simons and Vaz, 2004; Owen et al., 2006). These results are in perfect concordance with previous observations for di-4-ANEPPDHQ in HEK293 cells, where longer lifetimes were obtained in the plasma membrane in opposition to intracellular membranes (Owen et al., 2006). Similarly, the labeling of mammalian, *Drosophila* Kc and *Dictyostelium* AX2 cells with Laurdan probes also revealed a lower polarity of the plasma membrane lipid environment in contrast to intracellular membranes (Mazeres et al., 2017).

Interestingly, in CHO-K1 cells <τ> of di-4-ANEPPS was much longer (about 63%) than in *wt S. cerevisiae* plasma membrane (2.58 ± 0.07 ns), which is in very good agreement with the previously measured value in living *wt* yeast cells in mid-exponential phase in suspension (2.55 ± 0.06 ns) (Santos et al., 2017; Figures 4B,E). This may be compared with our observations in model membranes, where it was shown that Erg had a smaller ability to increase di-4-ANEPPS <τ> than Chol (Table 2). Interestingly, membrane organization as judged from <τ> of di-4-ANEPPS in vesicles containing either saturated PC or palmitoylsphingomyelin (PSM)/Chol lipids resembled most that of CHO-K1 cells, while membrane organization in POPC/Erg vesicles resembled that of *S. cerevisiae* cells. These results point to a fundamental difference in how to model sterol-enriched regions of mammalian and yeast cell membranes.

To study the influence of sterol profile on membrane properties reported by di-4-ANEPPS <τ>, *S. cerevisiae erg6*Δ cells were also analyzed, in which Erg is absent and the level of Zym is about four to seven-fold higher than in the *wt* strain (Zinser et al., 1993; Pedroso et al., 2009; Dupont et al., 2011). Despite the fact that Erg is no longer present, and Zym becomes one of the major cellular sterols (Heese-Peck et al., 2002; Guan et al., 2009; Dupont et al., 2011), no significant differences have been found in the levels of other major membrane lipids (Guan et al., 2009). At a first glance, FLIM images (Figures 4B,C) suggest a similar di-4-ANEPPS fluorescence lifetime in both *wt* and *erg6*Δ yeast strains. However, the frequency histograms (Figures 4E,F) point to a slightly longer <τ> of di-4-ANEPPS in *erg6*Δ cells (Supplementary Figure S4E; see also Supplementary Figure S7). This slight increase may be related to the higher Zym content of the mutant cells’ plasma membrane, as mentioned above. This would mean that in the presence of the main sterol of the plasma membrane in this strain, the probe would present a <τ> value similar to the one in the presence of Erg. In fact, that sterol is cholesta-5,7,24-trienol, which was found to accumulate in membrane regions resistant to detergent extraction in Erg6p defective mutants, at levels identical to those of Erg in *wt* cells (Eisenkolb et al., 2002).

**FIGURE 5:**
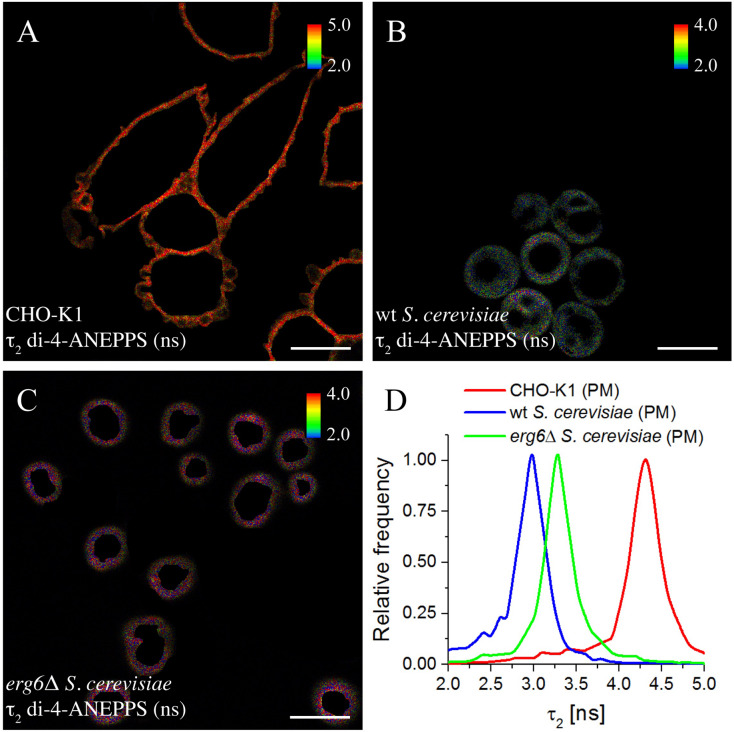
Long lifetime component τ_2_ of the fluorescence decay of di-4-ANEPPS is highly sensitive to changes in sterol-dependent properties of membranes. FLIM images of ROIs of the plasma membrane (PM) of CHO-K1 cells **(A)**, *wt* **(B)**, and *erg6*Δ *S. cerevisiae* cells **(C)**. The inset color bars give the long lifetime component τ_*2*_ range in ns used in the respective image. Scale bar = 10 μm. **(D)** Frequency histograms of τ_*2*_ corresponding to the FLIM images shown.

The fluorescence intensity decays of di-4-ANEPPS contain more information than the average fluorescence lifetime <τ>, namely the pre-exponentials and the fluorescence lifetimes of the two exponential components that describe each fluorescence intensity decay. These parameters were collected from all analyzed cells and the average values are given in Table 2. A noticeable difference is found in the value of τ_2_ between *wt* and *erg6*Δ, despite the small difference between <τ> of the probe in those yeast strains – τ_2_*_*wt*_* = 2.95 ns and τ_2_*_*erg*__6_*_Δ_ = 3.60 ns (Figure 5). The mutant strain value is more similar to the one obtained in model membranes containing Zym than Erg (Table 2). Comparing the lifetimes measured in yeast to those measured in CHO cells, both τ_2_ and <τ> were clearly longer in the mammalian cells, as in the model systems containing Chol compared to Erg and Zym.

Cell membranes are highly complex and dynamic, and their large number of components could conceivably influence di-4-ANEPPS measurements. Nevertheless, a good parallel could be drawn between the measured fluorescence lifetime components and amplitudes in mammalian and yeast cells of varied sterol profile and the model systems of closer sterol composition.

## Discussion

Recent studies in both yeast and mammalian cells have challenged the hypothesis that the main role of sterols is solely to generate *l*_d_/*l*_o_ lateral membrane heterogeneity (Souza et al., 2011). FLIM results and the high sterol content of eukaryotic plasma membranes suggest that an *l*_o_-like phase may actually correspond to a major area of the cell membrane (Owen et al., 2006; De Almeida and Joly, 2014; Kilin et al., 2015). These diverse perspectives can be reconciled by hypothesizing that while not all sterols are able to form *l*_o_ phase, the ability to form such phases in membrane model systems seems to be a common underlying feature of the main sterols of eukaryotic organisms. Here, we tested this hypothesis by a biophysical approach, in which physiologically relevant membrane properties in model systems containing the fungal and mammalian major sterols, Erg and Chol, were assessed. The measurements obtained with a probe suitable to report on sterol-dependent properties were directly compared to those performed in cell membranes of yeast and mammalian cells having different plasma membrane sterol profiles. Moreover, we made a parallel study of the last common biosynthetic precursor to Erg and Chol in the Blöch pathway, Zym.

The formation of *l*_o_ phase, a feature common to Erg and Chol, is a determining factor in lateral lipid organization of model membranes used in the present study. Previous observations on lipid monolayers upon sterol incorporation showed a steep increase of both DPPC and PSM monolayer condensation up to ≈ 30 mol% Chol, after which a plateau is reached (Hac-Wydro et al., 2014). At this composition, DPPC/Chol and PSM/Chol bilayers are 100% in *l*_o_ (Sankaram and Thompson, 1991; De Almeida et al., 2003). The plateau is not a result of the sterol solubility limit, i.e., for Chol in PC membranes this limit is much larger than 30 mol% (Huang et al., 1999; Mannock et al., 2010). For Zym, however, a slower and approximately linear increase in condensation was observed along all the composition range, i.e., up to 50 mol%, which can be explained by the inability of Zym to form a new phase analogous to the *l*_o_ phase found in lipid bilayer membranes. Those results for Zym-containing monolayers can be paralleled to the approximately linear increase in the <τ> of di-4-ANEPPS up to ca. 50 mol% of this sterol in binary mixtures with a fluid or gel PC presented in this work.

Crucial membrane biophysical features are imprinted by the major sterol component, one of the most important being, apparently, the ability to form an *l*_o_ phase or the lack of this ability. This feature has important consequences for biologically relevant properties, such as membrane passive permeability to water and univalent cations studied in this work. The hypersensitivity of Erg biosynthetic mutant cells to stress situations, such as dehydration (Dupont et al., 2011), univalent metal cations (Welihinda et al., 1994; Pagani et al., 2007; Ruotolo et al., 2008) and high saline conditions (Kodedova and Sychrova, 2015), could be related to the higher passive permeability of their cell membrane compared to the Erg-containing *wt* cells. In fact, increased permeability to small dyes such as Rhodamine 6G is a phenotypic feature of *erg6*Δ cells (Mukhopadhyay et al., 2002). Moreover, *erg6*Δ cells exhibit higher plasma membrane permeability to H_2_O_2_ and, thus, are more sensitive to this molecule (Branco et al., 2004), which can, at least in part, be explained by our observation that membranes lacking *l*_o_-forming sterols are more permeable to water and ions.

Previously, it has been hypothesized that sterols and sphingolipids have co-evolved to provide an optimal interaction between the two lipid groups (Guan et al., 2009). Although in mammalian cells sphingolipids and Chol are present in the same plasma membrane leaflet, in yeast there is evidence that Erg predominates in the inner leaflet whereas sphingolipids accumulate in the outer one. Thus, while in mammalian plasma membrane sphingolipid/Chol interactions are easily established in order to dominate the global properties of the membrane, in yeast plasma membrane Erg/sphingolipid interactions are not as likely to occur and therefore their contribution for the overall membrane properties seems more limited. Analysis of the double mutant yeast strain *elo3*Δ*erg6*Δ, with defects both in sphingolipid and sterol biosynthesis, revealed an important role of the methyl group at carbon 24 of the side chain of Erg for its interaction with sphingolipids (Eisenkolb et al., 2002). The Erg precursor Zym lacks this methyl group, but interestingly so does Chol. Thus, this methyl group should not be responsible for the driving force conferring Erg (and Chol) the ability to induce the formation of an *l*_o_ phase, but should be important for the stability of the fungal membrane. Conversely, the double bond at C-6 (Scheme 1), which is a common structural feature between Erg and Chol and absent in Zym, would, according to our results, likely be required for their *l*_o_-forming ability. On the other hand, a saturated acyl chain is a distinctive feature of Chol and could therefore be important for the significant increase in membrane dipole potential that only Chol could convey to the membrane (Figure 2). Thus, it appears that during the evolutionary process a fine-tuning of the structure of sterols, which confers e.g., variable tilt and orientation toward the acyl chains of phospholipids (Rog and Pasenkiewicz-Gierula, 2003; Rog et al., 2008), may have occurred, leading to the selection of Erg as the sterol that could preferentially associate with lipids present in yeast membranes, and Chol as the sterol that would perform a similar function in mammals. The observation that a yeast strain where Chol replaces Erg is even more sensitive to weak organic acids than *erg6*Δ (Souza et al., 2011) strengthens the hypothesis formulated above. In further support, Erg does not affect the di-4-ANEPPS <τ> in mixtures with PSM, whereas the longest <τ> was achieved when Chol was mixed with this same sphingolipid (Bastos et al., 2012), one of the most abundant in mammalian cells but absent in fungi.

Indeed, the probe di-4-ANEPPS is sensitive to sterol content and type, more than to membrane fluidity (Amaro et al., 2017). Factors influencing the fluorescence decay of di-4-ANEPPS include hydrogen bonds, both from hydration water and others mainly in the region of the phospholipid headgroups, which define the probe microenvironment (Loew, 1996; M’baye et al., 2008). Although some photophysical parameters of di-4-ANEPPS can be different between pure DPPC and POPC environments, the factors that govern the average fluorescence lifetime defined by the chemical environment of the probe are relatively similar, as the fluorophore is accommodated between the phosphocholine and the ester polar groups of the phospholipids (Celli and Gratton, 2010; Golfetto et al., 2013; Timr et al., 2014; Amaro et al., 2017). In fact, in PC/Chol mixtures, the general polarization values of di-4-ANEPPDHQ do not correlate with lipid packing as it is the case for Laurdan. Instead, they are influenced by the presence of Chol (Amaro et al., 2017). Thus, any changes in the di-4-ANEPPS <τ> in binary lipid bilayers of DPPC or POPC with increasing sterol concentrations are mostly attributed to sterol structural and dynamic features (Galván-Hernández et al., 2020) reflected on the membrane biophysical properties which of course, are also dependent on the lipid species interacting with the sterol (Bastos et al., 2012). Altogether, these features suggest that the probe reports preferentially the properties of sterol-enriched domains in more complex environments, such as that of a cellular membrane. In fact, the amplitude-weighted average fluorescence lifetime of di-4-ANEPPS was shown to correlate remarkably with the glycerophospholipid/Erg ratio along germination of *Neurospora crassa* asexual spores (Santos et al., 2018). Our study showed that di-4-ANEPPS fluorescence intensity decay parameters and <τ> in living cells can be recapitulated by membrane model systems of similar sterol composition. This happens for Chol containing membranes and CHO cells and for Erg-containing membrane and *wt* yeast cells. Moreover, the differences between *wt* and *erg6*Δ yeast cells can be related to the Zym ability to increase <τ> of the probe to a larger extent than Erg. However, these results also indicate that a longer <τ> of di-4-ANEPPS cannot be directly associated to the presence of *l*_o_ domains in yeast, as Zym is unable to induce the formation of this phase. The similarity between di-4-ANEPPS <τ> in *S. cerevisiae* cells and in POPC/Erg vesicles could indicate that the fraction of *l*_d_-like domains is higher in yeast than in CHO-K1 cells. However, several studies suggest that the plasma membrane of *S. cerevisiae* is considerably ordered, containing both *l*_o_ and gel domains (Klose et al., 2010; Aresta-Branco et al., 2011; Malinsky et al., 2013). Therefore, an alternative depiction of *S. cerevisiae* membrane organization that conciliates its ordered character with the relatively low di-4-ANEPPS <τ> is required. In multicomponent membranes, each domain type is enriched in certain lipids, but also contains minor fractions of other components. Thus, the *l*_o_ domains in yeast may contain a higher fraction of unsaturated phospholipids than their mammalian counterpart. On one hand, this hypothesis is consistent with the recent proposal that only ca. 20 mol% of Erg is localized in the outer leaflet of the plasma membrane (Solanko et al., 2018). Consequently, sphingolipids would be mainly forming Erg-depleted domains, whilst Erg-containing regions in the outer leaflet would largely correspond to a POPC/Erg (ca. 70:30 mole fraction) mixture, which is in fair agreement with the glycerophospholipid/Erg ratio in the outer leaflet calculated from the composition reported for the plasma membrane of *S*. *cerevisiae*, i.e., considering ca. 20 mol% Erg in the outer leaflet (Solanko et al., 2018). On the other hand this hypothesis also agrees with the fluorescence lifetime of di-4-ANEPPS in CHO-K1 cells, which indicates that in these cells sterol-enriched membrane regions are mainly formed by saturated lipids (sphingomyelin) and Chol. Indeed, the average lifetime <τ> obtained in the plasma membrane of CHO-K1 cells is identical to the ones observed for either DPPC/Chol or PSM/Chol (70:30 mole fraction), typical *l*_o_ phase forming mixtures, but not for POPC/Chol. In contrast, for *wt S. cerevisiae* cells, the membrane properties as reported by di-4-ANEPPS <τ> present closer similarity with POPC/Erg vesicles, rather than with DPPC/Erg. Consistently with these observations, the anisotropy of DPH in *wt* yeast cells (<*r*>∼ 0.15) (Aresta-Branco et al., 2011) is almost coincident with the one obtained in POPC/Erg mixtures containing more than 20 mol% Erg (<*r*>∼ 0.14) (Arora et al., 2004) whereas the higher anisotropy values determined in DPPC/Chol mixtures (Arora et al., 2004) correlate with those measured in CHO cells (Carreira, 2019). Taken together, these results support the seemingly different microdomain organization in yeast and animal cells: in yeast, a significant fraction of the saturated lipids are segregated into Erg-depleted gel domains that may act as diffusion barriers, stabilizing larger and less dynamic membrane compartments (Clay et al., 2014; Marques et al., 2015), while the Erg-rich *l*_o_ phase contains a large fraction of monounsaturated lipids; in mammalians, such gel domains, apart from cells with pathological levels of ceramide or glucosylceramide (Varela et al., 2014, 2017), have never been reported and saturated lipids are a major component of the Chol-rich phase.

## Conclusion

We compared the properties of Erg, Chol and Zym-containing membranes both in living cells and model membranes. This allowed us to extend our understanding of the importance of sterol-dependent membrane biophysical properties in eukaryotes. By establishing the ability to form *l*_o_ phase as the major common biophysical property of Erg and Chol that is not shared by Zym, our results may provide a general framework to a more insightful interpretation of observations both in model systems and cellular membranes.

As Zym is a biosynthetic precursor of both Chol and Erg, it is tempting to extrapolate our main conclusions to sterol selection during organism evolution. We hypothesize that sterol evolution in fungi and animals was divergent in terms e.g., of solvent dynamics and dipole potential, but was convergent in the *l*_o_ phase promoting aptitude. This model can be a useful framework for the interpretation of biological outcomes related to membrane-sterol alterations.

## Data Availability Statement

The datasets generated for this study are available on request to the corresponding author.

## Author Contributions

AK, JM, AB, CA, AB-O, SS, GL and RM performed the experiments and analyzed the data. AH and HM provided scientific and technical expertise to research, reviewed and edited the manuscript. HM and RA designed the project. AK, JM, AB, CA, SS, and RA designed the experimental work and wrote the manuscript. RA supervised and coordinated the research.

## Conflict of Interest

The authors declare that the research was conducted in the absence of any commercial or financial relationships that could be construed as a potential conflict of interest.

## References

[B1] Abe F. Hiraki T. (2009). Mechanistic role of ergosterol in membrane rigidity and cycloheximide resistance in *Saccharomyces cerevisiae*. *Biochim. Biophys. Acta* 1788 743–752. 10.1016/j.bbamem.2008.12.002 19118519

[B2] Amaro M. Reina F. Hof M. Eggeling C. Sezgin E. (2017). Laurdan and Di-4-ANEPPDHQ probe different properties of the membrane. *J. Phys. D Appl. Phys.* 50:134004. 10.1088/1361-6463/aa5dbc 29449744 PMC5802044

[B3] Aresta-Branco F. Cordeiro A. M. Marinho H. S. Cyrne L. Antunes F. De Almeida R. F. (2011). Gel domains in the plasma membrane of *Saccharomyces cerevisiae*: highly ordered, ergosterol-free, and sphingolipid-enriched lipid rafts. *J. Biol. Chem.* 286 5043–5054. 10.1074/jbc.m110.154435 21127065 PMC3037616

[B4] Arora A. Raghuraman H. Chattopadhyay A. (2004). Influence of cholesterol and ergosterol on membrane dynamics: a fluorescence approach. *Biochem. Biophys. Res. Commun.* 318 920–926. 10.1016/j.bbrc.2004.04.118 15147960

[B5] Athanasopoulos A. Andre B. Sophianopoulou V. Gournas C. (2019). Fungal plasma membrane domains. *FEMS Microbiol. Rev.* 43 642–673. 31504467 10.1093/femsre/fuz022

[B6] Barton D. H. Gunatilaka A. A. Jarman T. R. Widdowson D. A. Bard M. Woods R. A. (1975). Biosynthesis of terpenes and steroids. X. The sterols of some yeast mutants doubly defective in ergosterol biosynthesis. *J. Chem. Soc. Perkin.* 1 88–92. 10.1039/p19750000088 1094026

[B7] Bastos A. E. Marinho H. S. Cordeiro A. M. De Soure A. M. De Almeida R. F. (2012). Biophysical properties of ergosterol-enriched lipid rafts in yeast and tools for their study: characterization of ergosterol/phosphatidylcholine membranes with three fluorescent membrane probes. *Chem. Phys. Lipids* 165 577–588. 10.1016/j.chemphyslip.2012.06.002 22705749

[B8] Beattie M. E. Veatch S. L. Stottrup B. L. Keller S. L. (2005). Sterol structure determines miscibility versus melting transitions in lipid vesicles. *Biophys. J.* 89 1760–1768. 10.1529/biophysj.104.049635 15951379 PMC1366679

[B9] Bittman R. Blau L. (1976). Kinetics of solute permeability in phospholipid vesicles. *J. Chem. Educ.* 53 259–261. 943404 10.1021/ed053p259

[B10] Branco M. R. Marinho H. S. Cyrne L. Antunes F. (2004). Decrease of H2O2 plasma membrane permeability during adaptation to H2O2 in *Saccharomyces cerevisiae*. *J. Biol. Chem.* 279 6501–6506. 10.1074/jbc.m311818200 14645222

[B11] Carreira A. C. (2019). *Sphingosine-Induced Alterations in Membrane Biophysical Properties: Biological Relevance in the Pathophysiology of Human Disease.* Doctoral thesis, Universidade de Lisboa, Lisbon.

[B12] Celli A. Gratton E. (2010). Dynamics of lipid domain formation: fluctuation analysis. *Biochim. Biophys. Acta* 1798 1368–1376. 10.1016/j.bbamem.2009.12.002 20025848 PMC2883005

[B13] Clay L. Caudron F. Denoth-Lippuner A. Boettcher B. Buvelot Frei S. Snapp E. L. (2014). A sphingolipid-dependent diffusion barrier confines ER stress to the yeast mother cell. *eLife* 3:e01883. 10.7554/eLife.01883 PMC4009826 24843009

[B14] Clerc S. G. Thompson T. E. (1995). Permeability of dimyristoyl phosphatidylcholine/dipalmitoyl phosphatidylcholine bilayer membranes with coexisting gel and liquid-crystalline phases. *Biophys. J.* 68 2333–2341. 10.1016/s0006-3495(95)80415-7 7647237 PMC1282143

[B15] Cordeiro R. M. (2018). Molecular structure and permeability at the interface between phase-separated membrane domains. *J. Phys. Chem. B* 122 6954–6965. 10.1021/acs.jpcb.8b03406 29767519

[B16] Coutinho A. Silva L. Fedorov A. Prieto M. (2004). Cholesterol and ergosterol influence nystatin surface aggregation: relation to pore formation. *Biophys. J.* 87 3264–3276. 10.1529/biophysj.104.044883 15315952 PMC1304795

[B17] Daum G. Tuller G. Nemec T. Hrastnik C. Balliano G. Cattel L. (1999). Systematic analysis of yeast strains with possible defects in lipid metabolism. *Yeast* 15 601–614. 10.1002/(SICI)1097-0061(199905)15:7(601:AID-YEA390(3.0.CO;2-N 10341423

[B18] De Almeida R. F. Borst J. Fedorov A. Prieto M. Visser A. J. (2007). Complexity of lipid domains and rafts in giant unilamellar vesicles revealed by combining imaging and microscopic and macroscopic time-resolved fluorescence. *Biophys. J.* 93 539–553. 10.1529/biophysj.106.098822 17449668 PMC1896224

[B19] De Almeida R. F. Fedorov A. Prieto M. (2003). Sphingomyelin/phosphatidylcholine/cholesterol phase diagram: boundaries and composition of lipid rafts. *Biophys. J.* 85 2406–2416. 10.1016/s0006-3495(03)74664-5 14507704 PMC1303465

[B20] De Almeida R. F. Joly E. (2014). Crystallization around solid-like nanosized docks can explain the specificity, diversity, and stability of membrane microdomains. *Front. Plant Sci.* 5:72. 10.3389/fpls.2014.00072 24634670 PMC3943355

[B21] De Almeida R. F. Loura L. M. Fedorov A. Prieto M. (2005). Lipid rafts have different sizes depending on membrane composition: a time-resolved fluorescence resonance energy transfer study. *J. Mol. Biol.* 346 1109–1120. 10.1016/j.jmb.2004.12.026 15701521

[B22] Dietrich C. Bagatolli L. A. Volovyk Z. N. Thompson N. L. Levi M. Jacobson K. (2001). Lipid rafts reconstituted in model membranes. *Biophys. J.* 80 1417–1428. 10.1016/s0006-3495(01)76114-0 11222302 PMC1301333

[B23] Dupont S. Beney L. Ferreira T. Gervais P. (2011). Nature of sterols affects plasma membrane behavior and yeast survival during dehydration. *Biochim. Biophys. Acta* 1808 1520–1528. 10.1016/j.bbamem.2010.11.012 21081111

[B24] Eisenkolb M. Zenzmaier C. Leitner E. Schneiter R. (2002). A specific structural requirement for ergosterol in long-chain fatty acid synthesis mutants important for maintaining raft domains in yeast. *Mol. Biol. Cell* 13 4414–4428. 10.1091/mbc.e02-02-0116 12475962 PMC138643

[B25] Emter R. Heese-Peck A. Kralli A. (2002). ERG6 and PDR5 regulate small lipophilic drug accumulation in yeast cells via distinct mechanisms. *FEBS Lett.* 521 57–61. 10.1016/s0014-5793(02)02818-1 12067726

[B26] Folmer V. Pedroso N. Matias A. C. Lopes S. C. Antunes F. Cyrne L. (2008). H2O2 induces rapid biophysical and permeability changes in the plasma membrane of *Saccharomyces cerevisiae*. *Biochim. Biophys. Acta* 1778 1141–1147. 10.1016/j.bbamem.2007.12.008 18187036

[B27] Galván-Hernández A. Kobayashi N. Hernández-Cobos J. Antillón A. Nakabayashi S. Ortega-Blake I. (2020). Morphology and dynamics of domains in ergosterol or cholesterol containing membranes. *Biochim. Biophys. Acta* 1862:183101. 10.1016/j.bbamem.2019.183101 31672540

[B28] Golfetto O. Hinde E. Gratton E. (2013). Laurdan fluorescence lifetime discriminates cholesterol content from changes in fluidity in living cell membranes. *Biophys. J.* 104 1238–1247. 10.1016/j.bpj.2012.12.057 23528083 PMC3602759

[B29] Grossmann G. Opekarova M. Malinsky J. Weig-Meckl I. Tanner W. (2007). Membrane potential governs lateral segregation of plasma membrane proteins and lipids in yeast. *EMBO J.* 26 1–8. 10.1038/sj.emboj.7601466 17170709 PMC1782361

[B30] Guan X. L. Souza C. M. Pichler H. Dewhurst G. Schaad O. Kajiwara K. (2009). Functional interactions between sphingolipids and sterols in biological membranes regulating cell physiology. *Mol. Biol. Cell* 20 2083–2095. 10.1091/mbc.e08-11-1126 19225153 PMC2663937

[B31] Hac-Wydro K. Wydro P. Flasinski M. (2014). The comparison of zymosterol vs cholesterol membrane properties–the effect of zymosterol on lipid monolayers. *Colloids Surf. B Biointerfaces* 123 524–532. 10.1016/j.colsurfb.2014.09.054 25444659

[B32] Haldar S. Kanaparthi R. K. Samanta A. Chattopadhyay A. (2012). Differential effect of cholesterol and its biosynthetic precursors on membrane dipole potential. *Biophys. J.* 102 1561–1569. 10.1016/j.bpj.2012.03.004 22500756 PMC3318132

[B33] Heese-Peck A. Pichler H. Zanolari B. Watanabe R. Daum G. Riezman H. (2002). Multiple functions of sterols in yeast endocytosis. *Mol. Biol. Cell* 13 2664–2680. 10.1091/mbc.e02-04-0186 12181337 PMC117933

[B34] Hsueh Y. W. Chen M. T. Patty P. J. Code C. Cheng J. Frisken B. J. (2007). Ergosterol in POPC membranes: physical properties and comparison with structurally similar sterols. *Biophys. J.* 92 1606–1615. 10.1529/biophysj.106.097345 17142279 PMC1796827

[B35] Hsueh Y. W. Gilbert K. Trandum C. Zuckermann M. Thewalt J. (2005). The effect of ergosterol on dipalmitoylphosphatidylcholine bilayers: a deuterium NMR and calorimetric study. *Biophys. J.* 88 1799–1808. 10.1529/biophysj.104.051375 15596499 PMC1305234

[B36] Huang J. Buboltz J. T. Feigenson G. W. (1999). Maximum solubility of cholesterol in phosphatidylcholine and phosphatidylethanolamine bilayers. *Biochim. Biophys. Acta* 1417 89–100. 10.1016/s0005-2736(98)00260-0 10076038

[B37] Ipsen J. H. Mouritsen O. G. Bloom M. (1990). Relationships between lipid membrane area, hydrophobic thickness, and acyl-chain orientational order. The effects of cholesterol. *Biophys. J.* 57 405–412. 10.1016/s0006-3495(90)82557-1 2306491 PMC1280735

[B38] Johnson I. Spence M. T. Z. (2010). The Molecular probes handbook - a guide to fluorescent probes and labeling technologies. 11th edition, *Life Technologies*, 480–600.

[B39] Kaur R. Bachhawat A. K. (1999). The yeast multidrug resistance pump, Pdr5p, confers reduced drug resistance in erg mutants of *Saccharomyces cerevisiae*. *Microbiology* 145(Pt 4) 809–818. 10.1099/13500872-145-4-809 10220160

[B40] Khmelinskaia A. Ibarguren M. De Almeida R. F. M. Lopez D. J. Paixao V. A. Ahyayauch H. (2014). Changes in membrane organization upon spontaneous insertion of 2-hydroxylated unsaturated fatty acids in the lipid bilayer. *Langmuir* 30 2117–2128. 10.1021/la403977f 24490728

[B41] Kilin V. Glushonkov O. Herdly L. Klymchenko A. Richert L. Mely Y. (2015). Fluorescence lifetime imaging of membrane lipid order with a ratiometric fluorescent probe. *Biophys. J.* 108 2521–2531. 10.1016/j.bpj.2015.04.003 25992730 PMC4457243

[B42] Kirsch S. A. Böckmann R. A. (2019). Coupling of membrane nanodomain formation and enhanced electroporation near phase transition. *Biophys. J.* 116 2131–2148. 10.1016/j.bpj.2019.04.024 31103234 PMC6554532

[B43] Klose C. Ejsing C. S. Garcia-Saez A. J. Kaiser H. J. Sampaio J. L. Surma M. A. (2010). Yeast lipids can phase-separate into micrometer-scale membrane domains. *J. Biol. Chem.* 285 30224–30232. 10.1074/jbc.m110.123554 20647309 PMC2943255

[B44] Kodedova M. Sychrova H. (2015). Changes in the sterol composition of the plasma membrane affect membrane potential, salt tolerance and the activity of multidrug resistance pumps in *Saccharomyces cerevisiae*. *PLoS One* 10:e0139306. 10.1371/journal.pone.0139306 26418026 PMC4587746

[B45] Loew L. M. (1996). Potentiometric dyes: imaging electrical activity of cell membranes. *Pure Appl. Chem.* 68 1405–1409. 10.1351/pac199668071405

[B46] Loew L. M. Cohen L. B. Dix J. Fluhler E. N. Montana V. Salama G. (1992). A naphthyl analog of the aminostyryl pyridinium class of potentiometric membrane dyes shows consistent sensitivity in a variety of tissue, cell, and model membrane preparations. *J. Membr. Biol.* 130 1–10. 1469705 10.1007/BF00233734

[B47] Malinsky J. Opekarova M. (2016). New insight into the roles of membrane microdomains in physiological activities of fungal cells. *Int. Rev. Cell Mol. Biol.* 325 119–180. 10.1016/bs.ircmb.2016.02.005 27241220

[B48] Malinsky J. Opekarova M. Grossmann G. Tanner W. (2013). Membrane microdomains, rafts, and detergent-resistant membranes in plants and fungi. *Annu. Rev. Plant Biol.* 64 501–529. 10.1146/annurev-arplant-050312-120103 23638827

[B49] Mannock D. A. Lewis R. Mcelhaney R. N. (2010). A calorimetric and spectroscopic comparison of the effects of ergosterol and cholesterol on the thermotropic phase behavior and organization of dipalmitoylphosphatidylcholine bilayer membranes. *Biochim. Biophys. Acta* 1798 376–388. 10.1016/j.bbamem.2009.09.002 19761759

[B50] Marques J. T. Cordeiro A. M. Viana A. S. Herrmann A. Marinho H. S. De Almeida R. F. M. (2015). Formation and properties of membrane-ordered domains by phytoceramide: role of sphingoid base hydroxylation. *Langmuir* 31 9410–9421. 10.1021/acs.langmuir.5b02550 26262576

[B51] Mazeres S. Fereidouni F. Joly E. (2017). Using spectral decomposition of the signals from laurdan-derived probes to evaluate the physical state of membranes in live cells. *F1000Res* 6:763. 10.12688/f1000research.11577.2 28663788 PMC5473435

[B52] M’baye G. Mely Y. Duportail G. Klymchenko A. S. (2008). Liquid ordered and gel phases of lipid bilayers: fluorescent probes reveal close fluidity but different hydration. *Biophys. J.* 95 1217–1225. 10.1529/biophysj.107.127480 18390604 PMC2479600

[B53] McClare C. W. (1971). An accurate and convenient organic phosphorus assay. *Anal. Biochem.* 39 527–530. 10.1016/0003-2697(71)90443-x 4324534

[B54] Megha Bakht O. London E. (2006). Cholesterol precursors stabilize ordinary and ceramide-rich ordered lipid domains (lipid rafts) to different degrees. Implications for the Bloch hypothesis and sterol biosynthesis disorders. *J. Biol. Chem.* 281 21903–21913. 10.1074/jbc.m600395200 16735517

[B55] Mollapour M. Fong D. Balakrishnan K. Harris N. Thompson S. Schuller C. (2004). Screening the yeast deletant mutant collection for hypersensitivity and hyper-resistance to sorbate, a weak organic acid food preservative. *Yeast* 21 927–946. 10.1002/yea.1141 15334557

[B56] Mukhopadhyay K. Kohli A. Prasad R. (2002). Drug susceptibilities of yeast cells are affected by membrane lipid composition. *Antimicrob. Agents Chemother.* 46 3695–3705. 10.1128/aac.46.12.3695-3705.2002 12435664 PMC132749

[B57] Munn A. L. Heese-Peck A. Stevenson B. J. Pichler H. Riezman H. (1999). Specific sterols required for the internalization step of endocytosis in yeast. *Mol. Biol. Cell* 10 3943–3957. 10.1091/mbc.10.11.3943 10564282 PMC25690

[B58] Owen D. M. Lanigan P. M. P. Dunsby C. Munro I. Grant D. Neil M. A. A. (2006). Fluorescence lifetime imaging provides enhanced contrast when imaging the phase-sensitive dye di-4-ANEPPDHQ in model membranes and live cells. *Biophys. J.* 90 L80–L82. 16617080 10.1529/biophysj.106.084673 PMC1459501

[B59] Pagani M. A. Casamayor A. Serrano R. Atrian S. Arino J. (2007). Disruption of iron homeostasis in *Saccharomyces cerevisiae* by high zinc levels: a genome-wide study. *Mol. Microbiol.* 65 521–537. 10.1111/j.1365-2958.2007.05807.x 17630978

[B60] Pedroso N. Matias A. C. Cyrne L. Antunes F. Borges C. Malho R. (2009). Modulation of plasma membrane lipid profile and microdomains by H2O2 in *Saccharomyces cerevisiae*. *Free Radic. Biol. Med.* 46 289–298. 10.1016/j.freeradbiomed.2008.10.039 19027845

[B61] Rog T. Pasenkiewicz-Gierula M. (2003). Effects of epicholesterol on the phosphatidylcholine bilayer: a molecular simulation study. *Biophys. J.* 84 1818–1826. 10.1016/s0006-3495(03)74989-3 12609883 PMC1302750

[B62] Rog T. Vattulainen I. Jansen M. Ikonen E. Karttunen M. (2008). Comparison of cholesterol and its direct precursors along the biosynthetic pathway: effects of cholesterol, desmosterol and 7-dehydrocholesterol on saturated and unsaturated lipid bilayers. *J. Chem. Phys.* 129:154508 10.1063/1.2996296 19045210

[B63] Ruotolo R. Marchini G. Ottonello S. (2008). Membrane transporters and protein traffic networks differentially affecting metal tolerance: a genomic phenotyping study in yeast. *Genome Biol.* 9 R67. 10.1186/gb-2008-9-4-r67 PMC2643938 18394190

[B64] Sankaram M. B. Thompson T. E. (1991). Cholesterol-induced fluid-phase immiscibility in membranes. *Proc. Natl. Acad. Sci. U.S.A.* 88 8686–8690. 10.1073/pnas.88.19.8686 1656453 PMC52574

[B65] Santos F. C. Fernandes A. S. Antunes C. A. C. Moreira F. P. Videira A. Marinho H. S. (2017). Reorganization of plasma membrane lipid domains during conidial germination. *Biochim. Biophys. Acta* 1862 156–166. 10.1016/j.bbalip.2016.10.011 27815222

[B66] Santos F. C. Lobo G. M. Fernandes A. S. Videira A. De Almeida R. F. M. (2018). Changes in the biophysical properties of the cell membrane are involved in the response of Neurospora crassa to staurosporine. *Front. Physiol.* 9:1375. 10.3389/fphys.2018.01375 30364194 PMC6193110

[B67] Scheidt H. A. Meyer T. Nikolaus J. Baek D. J. Haralampiev I. Thomas L. (2013). Cholesterol’s aliphatic side chain modulates membrane properties. *Angew Chem.* 52 12848–12851. 10.1002/anie.201306753 24382636 PMC4011182

[B68] Scolari S. Engel S. Krebs N. Plazzo A. P. De Almeida R. F. Prieto M. (2009). Lateral distribution of the transmembrane domain of influenza virus hemagglutinin revealed by time-resolved fluorescence imaging. *J. Biol. Chem.* 284 15708–15716. 10.1074/jbc.m900437200 19349276 PMC2708868

[B69] Sezgin E. Levental I. Mayor S. Eggeling C. (2017). The mystery of membrane organization: composition, regulation and roles of lipid rafts. *Nat. Rev. Mol. Cell Biol.* 18 361–374. 10.1038/nrm.2017.16 28356571 PMC5500228

[B70] Silva L. Coutinho A. Fedorov A. Prieto M. (2006). Competitive binding of cholesterol and ergosterol to the polyene antibiotic nystatin. A fluorescence study. *Biophys. J.* 90 3625–3631. 10.1529/biophysj.105.075408 16500971 PMC1440743

[B71] Simons K. Vaz W. L. (2004). Model systems, lipid rafts, and cell membranes. *Annu. Rev. Biophys. Biomol. Struct.* 33 269–295. 10.1146/annurev.biophys.32.110601.141803 15139814

[B72] Solanko L. M. Sullivan D. P. Sere Y. Y. Szomek M. Lunding A. Solanko K. A. (2018). Ergosterol is mainly located in the cytoplasmic leaflet of the yeast plasma membrane. *Traffic* 19 198–214. 10.1111/tra.12545 29282820

[B73] Souza C. M. Schwabe T. M. Pichler H. Ploier B. Leitner E. Guan X. L. (2011). A stable yeast strain efficiently producing cholesterol instead of ergosterol is functional for tryptophan uptake, but not weak organic acid resistance. *Metab. Eng.* 13 555–569. 10.1016/j.ymben.2011.06.006 21741494

[B74] Steck T. L. Lange Y. (2018). Transverse distribution of plasma membrane bilayer cholesterol: Picking sides. *Traffic* 19 750–760. 10.1111/tra.12586 29896788

[B75] Stockl M. Nikolaus J. Herrmann A. (2010). Visualization of lipid domain-specific protein sorting in giant unilamellar vesicles. *Methods Mol. Biol.* 606 115–126. 10.1007/978-1-60761-447-0_10 20013394

[B76] Subczynski W. K. Hyde J. S. Kusumi A. (1989). Oxygen permeability of phosphatidylcholine–cholesterol membranes. *Proc. Natl. Acad. Sci. U.S.A.* 86 4474–4478. 10.1073/pnas.86.12.4474 2543978 PMC287292

[B77] Timr S. Bondar A. Cwiklik L. Stefl M. Hof M. Vazdar M. (2014). Accurate determination of the orientational distribution of a fluorescent molecule in a phospholipid membrane. *J. Phys. Chem. B* 118 855–863. 10.1021/jp4067026 24261603

[B78] Valachovic M. Bareither B. M. Shah Alam B. M. Eckstein J. Barbuch R. Balderes D. (2006). Cumulative mutations affecting sterol biosynthesis in the yeast *Saccharomyces cerevisiae* result in synthetic lethality that is suppressed by alterations in sphingolipid profiles. *Genetics* 173 1893–1908. 10.1534/genetics.105.053025 16702413 PMC1569731

[B79] Varela A. R. P. Da Silva A. Fedorov A. Futerman A. H. Prieto M. Silva L. C. (2014). Influence of intracellular membrane pH on sphingolipid organization and membrane biophysical properties. *Langmuir* 30 4094–4104. 10.1021/la5003397 24654655

[B80] Varela A. R. P. Ventura A. E. Carreira A. C. Fedorov A. Futerman A. H. Prieto M. (2017). Pathological levels of glucosylceramide change the biophysical properties of artificial and cell membranes. *Phys. Chem. Chem. Phys.* 19 340–346. 10.1039/c6cp07227e 27905603

[B81] Veatch S. L. Keller S. L. (2003). Separation of liquid phases in giant vesicles of ternary mixtures of phospholipids and cholesterol. *Biophys. J.* 85 3074–3083. 10.1016/s0006-3495(03)74726-2 14581208 PMC1303584

[B82] Vist M. R. Davis J. H. (1990). Phase equilibria of cholesterol/dipalmitoylphosphatidylcholine mixtures: 2H nuclear magnetic resonance and differential scanning calorimetry. *Biochemistry* 29 451–464. 10.1021/bi00454a021 2302384

[B83] Welihinda A. A. Beavis A. D. Trumbly R. J. (1994). Mutations in LIS1 (ERG6) gene confer increased sodium and lithium uptake in *Saccharomyces cerevisiae*. *Biochim. Biophys. Acta* 1193 107–117. 10.1016/0005-2736(94)90339-5 8038180

[B84] Zahumensky J. Malinsky J. (2019). Role of MCC/eisosome in fungal lipid homeostasis. *Biomolecules* 9:305. 10.3390/biom9080305 31349700 PMC6723945

[B85] Zinser E. Paltauf F. Daum G. (1993). Sterol composition of yeast organelle membranes and subcellular distribution of enzymes involved in sterol metabolism. *J. Bacteriol.* 175 2853–2858. 10.1128/jb.175.10.2853-2858.1993 8491706 PMC204601

