# Supplementary material for: Liquid-Ordered Phase Formation by Mammalian and Yeast Sterols: A Common Feature With Organizational Differences
Source: Front Cell Dev Biol. 2020 Jun 12;8:337. doi: 10.3389/fcell.2020.00337 (PMC7304482; doi:10.3389/fcell.2020.00337)
Supplement: Supplementary file 1 [file Data_Sheet_1.pdf]

Supplementary Material

Liquid-ordered phase in mammalian and yeast membranes: a common feature with organizational differences

Alena Khmelinskaia, Joaquim M. T. Marquês, André E.P. Bastos, Catarina A. C. Antunes, Andreia Bento-Oliveira, Silvia Scolari, Gerson M. da S. Lobo, Rui Malhó, Andreas Herrmann, H. Susana Marinho, Rodrigo F. M. de Almeida

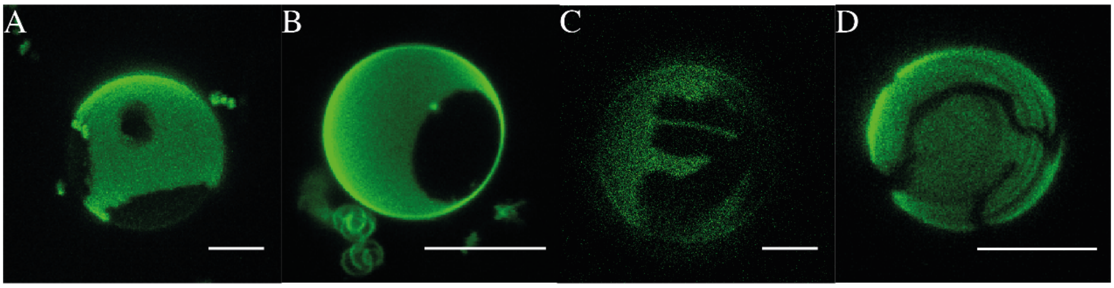

Supplementary Figure S1 – Confocal microscopy images of GUVs of (A) DOPC/DPPC/Chol, (B) DOPC/DPPC/Erg and (C) DOPC/DPPC/Zym (1:1:1 molar ratio), and of (D) DOPC/DPPC/Zym (1:1:2 molar ratio) at 23 ºC, labeled with Rhod-DOPE (0.2 mol%). Scale bar = 5 µm.

Supplementary Note 1 – Membrane passive permeability to water: additional methods

The absorbance values at time zero were confirmed by adding the same volume of liposome suspension to isotonic buffer. The normalized median of each set of triplicates was used for graphical comparison (Supplementary Figure S2). For a quantitative comparison, $\Delta(1/Abs_{550\,nm})/min$ (which is proportional to the osmotic water permeability [Lande et al. 1995]) was calculated from the initial slope of the curves (first minute) – Table 1.

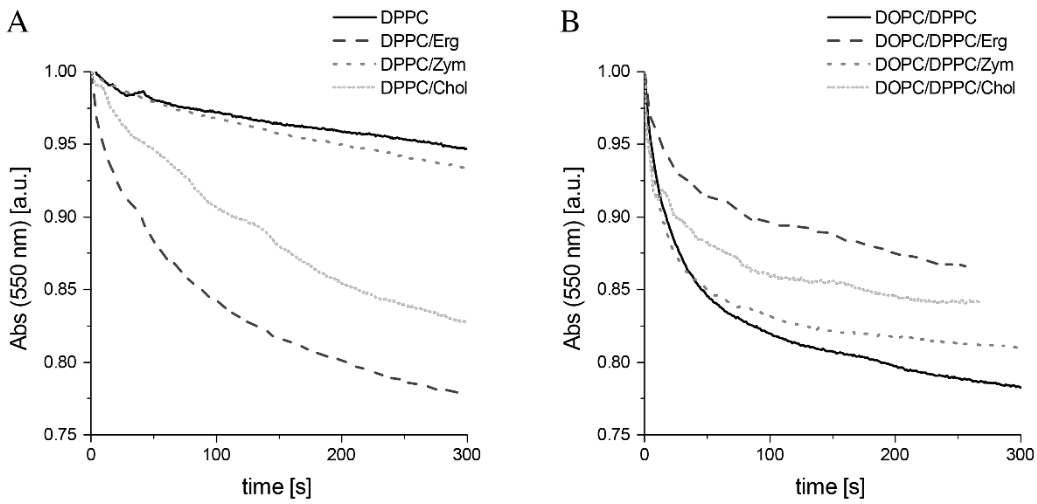

Supplementary Figure S2 – Membrane passive permeability to water. Representative absorption curves at 550 nm of DPPC (A) and DPPC/DOPC (1:1 molar ratio) (B) MLVs containing, or not, an equimolar proportion of each studied sterol, Erg, Zym or Chol. Curves were obtained by MLV transfer to hypotonic buffer. All experiments were performed at 24 ºC.

Supplementary Note 2 – Membrane passive permeability to potassium: additional methods

The vesicles were subjected to a transmembrane K^+ gradient of 4:1 $[K^+]_{in}/[K^+]_{out}$ through direct dilution of 0.33 mL of the previous lipid suspension into 1.0 mL of osmotically balanced S buffer (20 mM HEPES, pH 7.4, 230 mM sucrose, 3 mM NaN_3) in a magnetically stirred fluorescence quartz cuvette and the time course of the change in pyranine fluorescence intensity was followed at 510 nm (excitation at 460 nm) for 15 min. The final lipid concentration in solution was ≈ 1.3 mM. After this first time-course measurement, FCCP was added to the LUVs in a 1/460 FCCP-to-lipid fraction, to freely equilibrate the H^+ ions for another 15 min. At the end of the experiment, the fluorescence emission corresponding to total dissipation of the K^+ gradient was measured by adding the ionophore valinomycin (at 12.5 μM final concentration) to the lipid vesicles, as a control of the LUV integrity. Note that the fluorescence intensity of the entrapped pyranine is found to be linear with respect to pH for $7.0 < pH < 7.8$ [Coutinho et al. 2004].

The initial decrease in the fluorescence intensity was a consequence of K^+ leakage, due to a transmembrane K^+ gradient of 4:1 $[K^+]_{in}/[K^+]_{out}$ [Coutinho et al 2004], slowly accompanied by H^+ entrance. Although the membrane has a small permeability to both ions, it is considered that the relative membrane permeability is slightly higher for K^+ than H^+, and so, according to the *Goldman-Hodgkin-Katz* equation [Bowman and Baglioni 1984], H^+ inward movement across the membrane is the limiting factor for the outward movement of K^+ as a result of the K^+ concentration gradient. After 15 min, when the protonophore FCCP was added, a fast decrease in fluorescence intensity that reports the H^+ entrance was observed. In agreement with the *Goldman-Hodgkin-Katz* equation [Bowman and Baglioni 1984], the increase in the relative membrane permeability to H^+ promoted by FCCP insertion, leads to a fast H^+ entrance to compensate the initial K^+ leakage. After 15 min of equilibration, the K^+ ionophore, valinomycin, was added to the lipid vesicles, as a control of the LUV integrity. With the addition of valinomycin, a small increase of fluorescence intensity was observed. At this stage, there are channels for both ions, and the membrane is selectively permeable to both H^+ and K^+. Therefore, the membrane potential will not be at the equilibrium potential for any of the ions. So, in order to reach the membrane potential equilibrium condition after the incorporation of valinomycin, some protons will move outside of the liposomes.

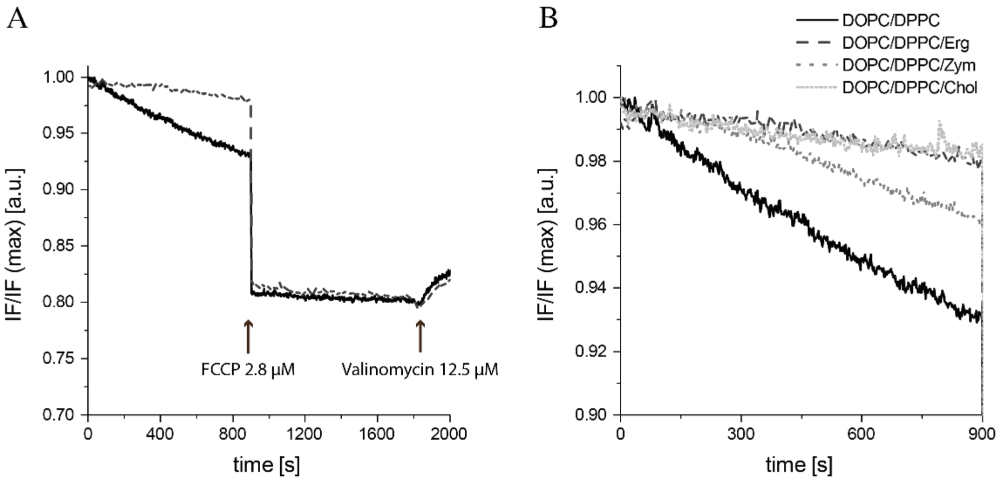

Supplementary Figure S3 – Membrane passive permeability to potassium. Representative normalized fluorescence intensity curves of pyranine at 510 nm in DPPC/DOPC (1:1 molar ratio) MLVs containing, or not, an equimolar proportion of each studied sterol (A). MLVs were subjected to a potassium gradient. (B) For a quantitative comparison between the sterols, the initial slope of the curves was calculated, as a measure of bilayer permeability to potassium (Table 1). All experiments were performed at 24 ºC.

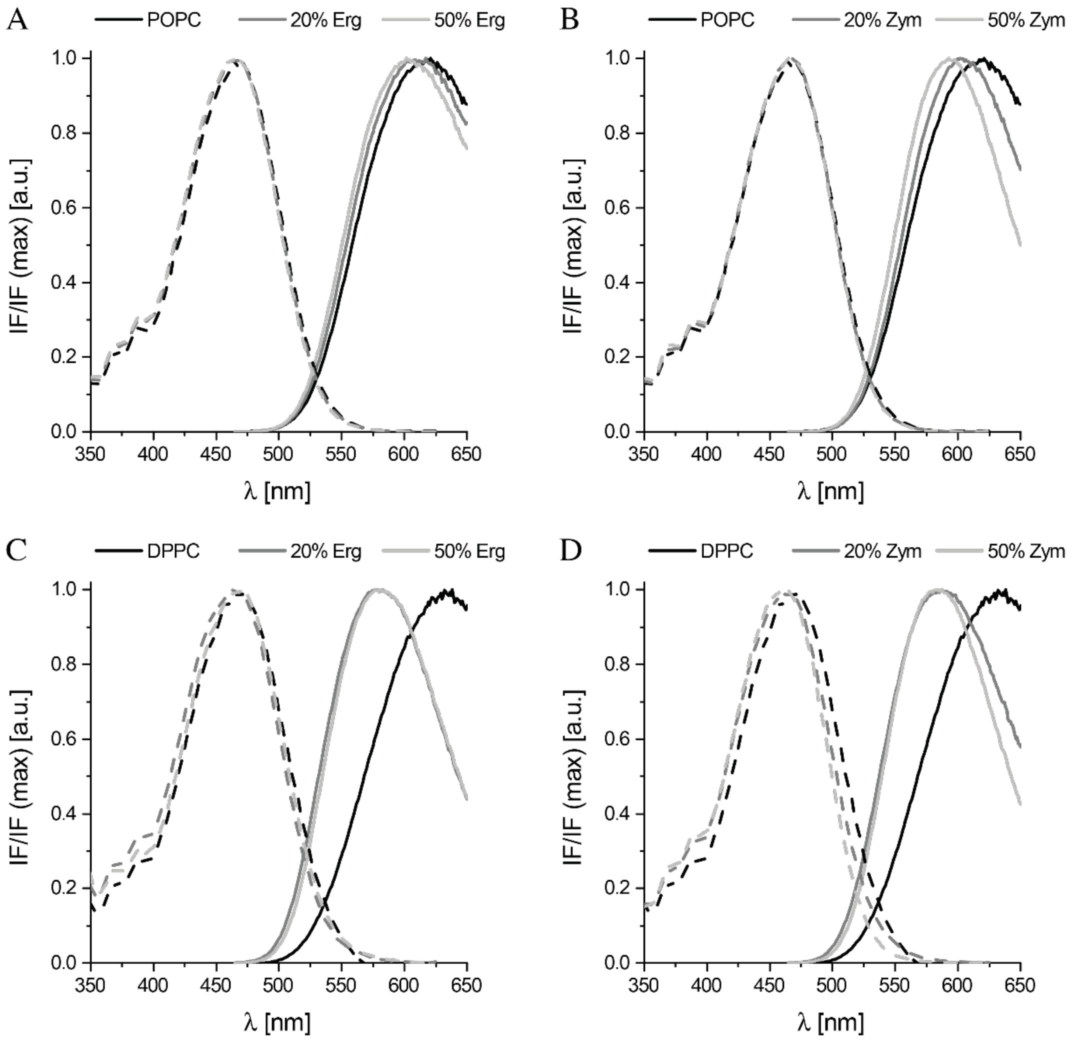

Supplementary Figure S4 – Median excitation (---) and emission (−) spectra of di-4-ANEPPS incorporated in POPC (A,B) and DPPC (C,D) MLVs containing different mol% of Erg (A,C) and Zym (B,D). Probe:lipid ratio 1:200. All experiments were performed at 24 ºC.

Supplementary Note 3 – Analysis of fluorescence lifetime microscopy imaging data

FLIM images were acquired for 60 s (45 frames with an average photon count rate of 2-4 × 10^4 count/s). Electrical signals were processed by the Time-Harp 200 PC card (PicoQuant). The fluorescence intensity decays were analyzed using the SymPhoTime software (PicoQuant), taking into account the instrument response function (IRF). At least, 50000 photons/cell were recorded. In Figure 4A-C of the main text, FLIM images of the three types of cells studied are shown. For each image, the plasma membranes (PM) and the intracellular membranes (IM) were separated into different regions of interest (ROI), as exemplified for one cell of each type in Supplementary Figure S5. The histograms of the pixel distribution of fluorescence lifetimes were reconstructed for each type of membrane (Figure 4D-F in the main text). For *S. cerevisiae* cells, the histogram analysis allowed to disclose a small difference between the fluorescence lifetime of di-4-ANEPPS in PM and IM. The measured photon/pixel retrieved from each image, pertaining to a given membrane type (PM or IM), were combined into a single decay curve shown in Supplementary Figure S6, to increase the total number of photons in each curve analyzed and illustrate the fluorescence intensity decay profiles for different types of membranes with very high signal-to-noise-ratio. The experimental decays were analyzed using a nonlinear least-squares iterative procedure, with a sum of two exponentials (Equation 1 in the main text, see Supplementary Table S1 for the retrieved parameters).

The fluorescence decay parameters presented in Table 2 of the main text for the different cell types and membranes (PM and IM) and the mean fluorescence lifetimes presented in Supplementary Figure S7 are the result of averaging all the values obtained by fitting the decays of the PM or IM of each individual cell to a double exponential model as described above and under the Materials and Methods in the main text.

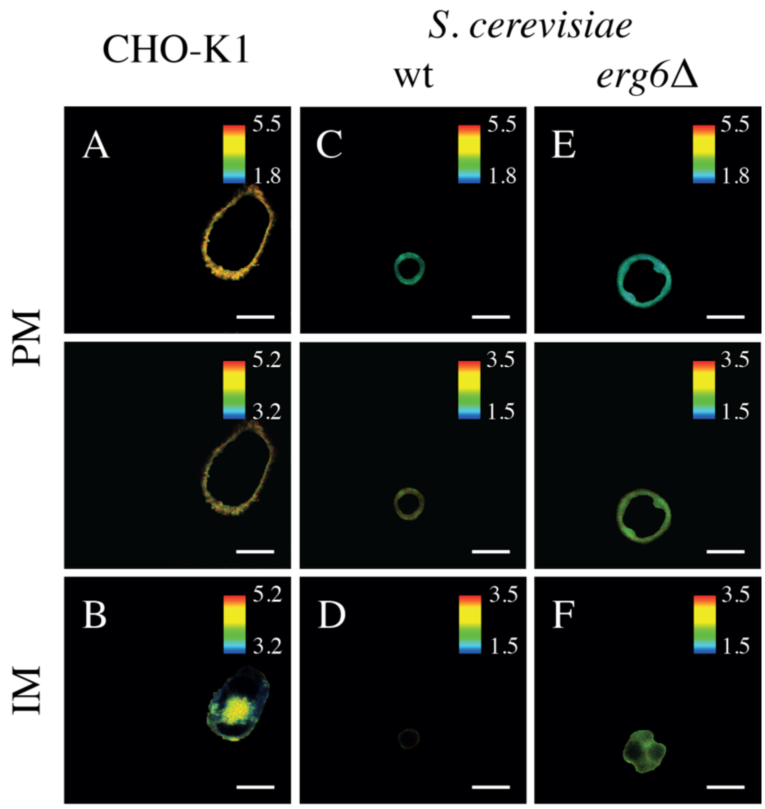

Supplementary Figure S5 –ROI selected for FLIM images of mammalian (CHO-K1), *S. cerevisiae wt* and *erg6*Δ cells, labeled with di-4-ANEPPS presented in Figure 4. The selected ROI correspond to the plasma membrane, PM (A, C, E) or intracellular membranes, IM (B, D, F) of a single cell. The mean fluorescence lifetime ⟨τ⟩ is shown as pseudocolor image in ns (see scale). For easier comparison, two different dynamic ranges are shown for the PM of each cell type. White scale bar = 10 μm.

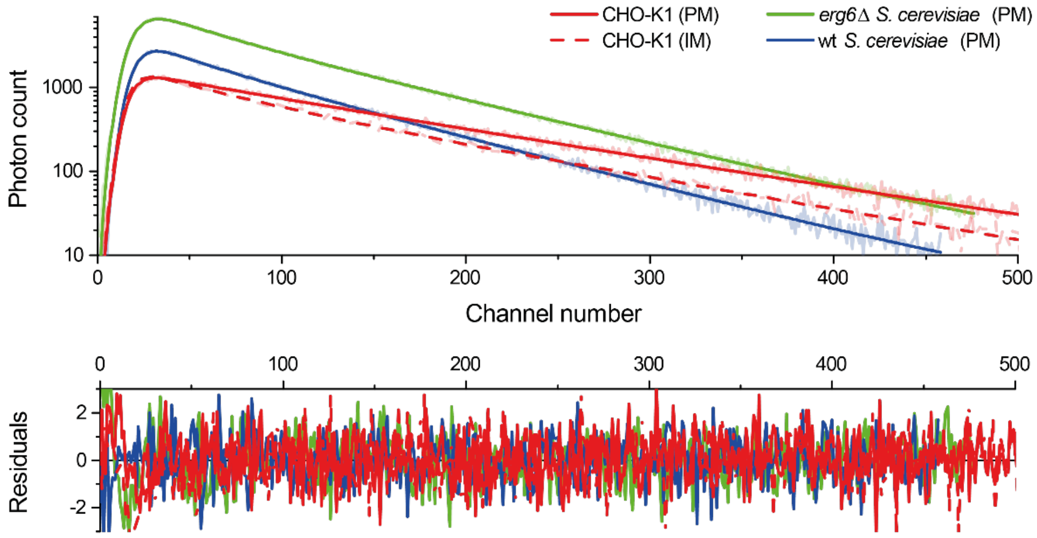

Supplementary Figure S6 – Experimental fluorescence intensity decays of di-4-ANEPPS retrieved by FLIM corresponding to the total photons emitted from selected ROI of the studied cell membrane types (see Figure 4 and Supplementary Figure S5), are shown. The bold lines correspond to the fitted bi-exponential model and the respective residuals are represented in the bottom panel. The retrieved parameters can be found in the Supplementary Table S1.

Supplementary Table S1 – Fluorescence intensity decay parameters that best describe the experimental decays shown on Supplementary Figure S6. The fluorescence intensity decays were described by the sum of two exponentials, with amplitudes α_1 and α_2 and lifetimes τ_1 and τ_2. $\bar{\tau}$ is the amplitude-weighted average fluorescence lifetime and $\langle\tau\rangle$ is the intensity-weighted mean fluorescence lifetime (Eqs. 4–6). χ^2 is the reduced-chi square of the fitting. PM stands for plasma membrane and IM for intracellular membranes.

**Cell type**	$\boldsymbol{\alpha_1}$	$\boldsymbol{\tau_1}$ **(ns)**	$\boldsymbol{\alpha_2}$	$\boldsymbol{\tau_2}$ **(ns)**	$\bar{\tau}$ **(ns)**	$\langle\tau\rangle$ **(ns)**	$\boldsymbol{\chi^2}$
CHO-K1 PM	0.157	2.142	0.843	4.457	4.09	4.27	1.07
CHO-K1 IM	3.371	1.482	0.629	3.952	3.03	3.50	1.18
S. cerevisiae wt PM	0.288	1.224	0.712	2.715	2.29	2.48	1.00
*S. cerevisiae erg6*Δ PM	0.381	1.486	0.619	3.024	2.44	2.67	1.17

Supplementary Note 4 – ⟨τ⟩ of di-4-ANEPPS is independent of membrane polarization

Since the spectral properties of potential sensitive dyes, such as di-4-ANEPPS, are usually affected by changes in the transmembrane potential [Loew et al. 1992; Laurita et al. 2001] it was important to ensure that under our conditions, the differences found in ⟨τ⟩ of di-4-ANEPPS were reporting only the sterol-dependent biophysical properties of the membrane. Therefore, we evaluated the effect of membrane potential loss in yeast cells induced by the protonophore CCCP on di-4-ANEPPS ⟨τ⟩, and determined that the fluorescence intensity decay of the probe remained unaltered when compared to non-treated yeast cells (Supplementary Figure S7C,E). Notably, the polarization state of yeast cells is known to be associated to changes in the sphingolipid-enriched gel domains, and not to Erg-rich l_o-like membrane regions [Herman et al. 2015]. Thus, the fact that ⟨τ⟩ of di-4-ANEPPS is not depending on the polarization state of membranes in living cells supports that it is reporting essentially on the sterol-dependent properties of the membrane, as already supported by the results in membrane model systems (Figure 3, Table 2).

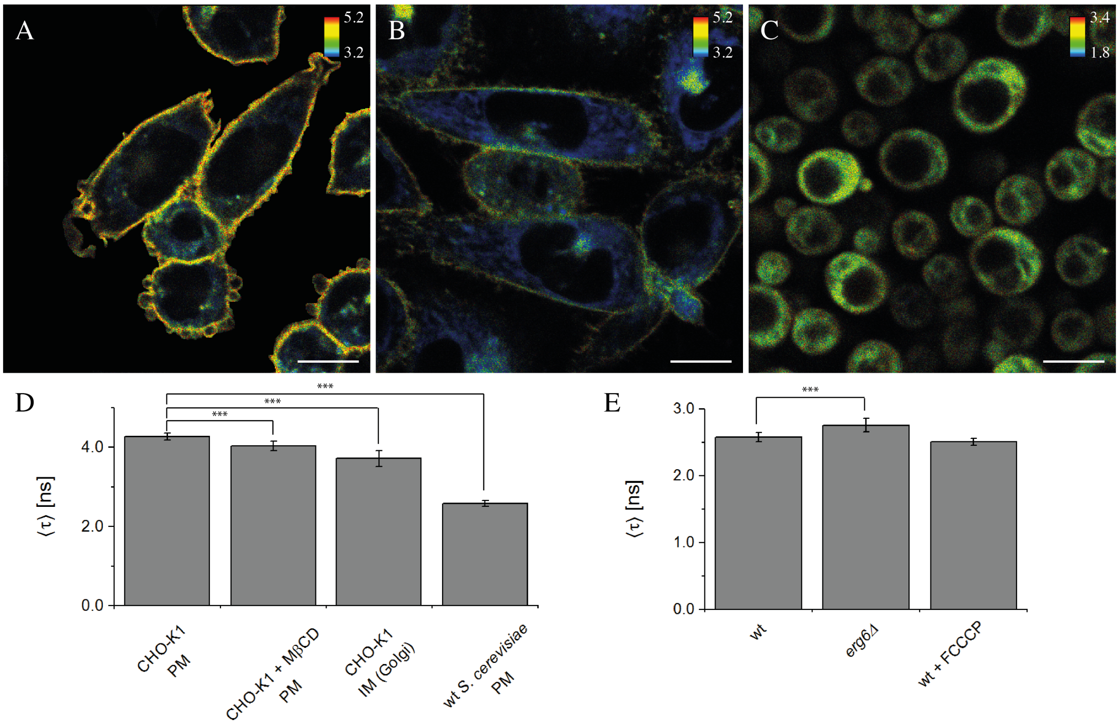

Supplementary Figure S7 – Fluorescence lifetime behavior of di-4-ANEPPS in mammalian and yeast cells is in agreement with that of model membranes. FLIM images of (A) untreated and (B) MβCD treated CHO-K1 cells, and (C) wt *S. cerevisiae* cells depolarized with CCCP. The mean fluorescence lifetime ⟨τ⟩ is shown as pseudocolor image in ns (see scale). White scale bar = 10 µm. D) Mean fluorescence lifetime ⟨τ⟩ of di-4-ANEPPS in the plasma membrane (PM) of untreated and MβCD treated CHO-K1 cells, in intracellular membranes (IM) of CHO-K1 cells and yeast plasma membrane was obtained from FLIM data analysis, each from at least 25 cells and at least two independent experiments in the case of CHO cells and at least 60 cells from at least three independent experiments in the case of yeast cells. E) Comparison of di-4-ANEPPS ⟨τ⟩ in *S. cerevisiae* cells plasma membrane: *wt*, *erg6*Δ and depolarized *wt* with CCCP. The values are the mean ± SD. Statistically significant differences were assessed through Student's t-test; *** $P < 0.001$.

Supplementary Note 5 – ⟨τ⟩ of di-4-ANEPPS can be correlated to membrane sterol levels *in vivo*

To test in a direct manner if ⟨τ⟩ of di-4-ANEPPS was sensitive to the Chol levels of the plasma membrane, CHO-K1 cells were subjected to a mild methyl-β-cyclodextrin (MβCD) treatment reducing their Chol content by ca. 12 % (see Materials and Methods). Indeed, MβCD treatment (Supplementary Figure S7B) led to a generalized decrease in ⟨τ⟩ of di-4-ANEPPS both in the intracellular membranes as well as in the plasma membrane when compared to untreated cells (Figure 4A, Supplementary Figure S7A): $\langle\tau\rangle = 4.03 \pm 0.12$ ns (treated) and $\langle\tau\rangle = 4.27 \pm 0.09$ ns (untreated) for the plasma membrane (Figure 4D). Considering that in DPPC bilayers the addition of Chol corresponds to a maximum increase of ⟨τ⟩ by ≈ 2.2 ns for di-4-ANEPPS (Figure 3B), the decrease of 0.24 ns between treated and untreated cells would correspond roughly to a removal of 11 % of Chol, in agreement with the 12 % ^{3}H-Chol obtained by scintillation counting [Scolari et al. 2009]. The decrease in ⟨τ⟩ we observed is about 0.4 – 0.5 ns smaller than the one observed by Owen et al in HEK293 cells using di-4-ANEPPDHQ [Owen et al. 2006], however the treatment conditions used by these authors lead to a larger decrease in Chol content. Thus, changes in ⟨τ⟩ of di-4-ANEPPS can be correlated to the Chol levels *in vivo*.

Additional references

Bowman, C.L. and Baglioni, A. Application of the Goldman-Hodgkin-Katz current equation to membrane current-voltage data. Journal of Theoretical Biology. 1984;108(1):1-29.

Coutinho, A., Silva, L., Fedorov, A., Prieto, M. Cholesterol and Ergosterol Influence Nystatin Surface Aggregation: Relation to Pore Formation. Biophysical Journal. 2004;87(5):3264–3276.

Herman P, Vecer J, Opekarova M, Vesela P, Jancikova I, Zahumensky J, et al. Depolarization affects the lateral microdomain structure of yeast plasma membrane. The FEBS journal. 2015;282(3):419-34.

Lande MB, Donovan JM, Zeidel ML. The relationship between membrane fluidity and permeabilities to water, solutes, ammonia, and protons. The Journal of general physiology. 1995;106(1):67-84.

Laurita KR, Singal A. Mapping action potentials and calcium transients simultaneously from the intact heart. American journal of physiology Heart and circulatory physiology. 2001;280(5):H2053-60.

Loew LM, Cohen LB, Dix J, Fluhler EN, Montana V, Salama G, et al. A naphthyl analog of the aminostyryl pyridinium class of potentiometric membrane dyes shows consistent sensitivity in a variety of tissue, cell, and model membrane preparations. The Journal of membrane biology. 1992;130(1):1-10.

Owen DM, Lanigan PM, Dunsby C, Munro I, Grant D, Neil MA, et al. Fluorescence lifetime imaging provides enhanced contrast when imaging the phase-sensitive dye di-4-ANEPPDHQ in model membranes and live cells. Biophysical journal. 2006;90(11):L80-82.

Scolari S, Engel S, Krebs N, Plazzo AP, De Almeida RF, Prieto M, et al. Lateral distribution of the transmembrane domain of influenza virus hemagglutinin revealed by time-resolved fluorescence imaging. The Journal of biological chemistry. 2009;284(23):15708-16.
